# Mechanically mutable polymer enabled by light

**DOI:** 10.1126/sciadv.abo1626

**Published:** 2022-08-24

**Authors:** Feng Cai, Bowen Yang, Xuande Lv, Wei Feng, Haifeng Yu

**Affiliations:** ^1^ School of Materials Science and Engineering, Key Laboratory of Polymer Chemistry and Physics of Ministry of Education, Peking University, Beijing 100871, P. R. China.; ^2^ School of Materials Science and Engineering, Tianjin Key Laboratory of Composite and Functional Materials, Tianjin University, Tianjin 300072, P. R. China.

## Abstract

Human skin is a remarkable example of a biological material that displays unique mechanical characters of both soft elasticity and stretchability. However, mimicking these features has been absent in photoresponsive soft matters. Here, we present one synthetic ABA-type triblock copolymer consisting of polystyrene as end blocks and one photoresponsive azopolymer as the middle block, which is stiffness at room temperature and shows a phototunable transition to soft elastics athermally. We have synthesized an elastics we term “photoinduced soft elastomer,” where the photo-evocable soft midblock of azopolymer and the glassy polystyrene domains act as elastic matrix and physical cross-linking junctions, respectively. On the basis of the photoswitchable transformation between stiffness and elasticity at room temperature, we demonstrated precise control over nanopatterns on nonplanar substrates especially adaptable in the human skin and fabrication of packaged perovskite solar cells, enabling the simple, human-friendly, and controllable approach to be promising for mechanically adaptable soft photonic and electronic packaging applications.

## Teaser

A mechanically mutable material with physical cross-linking and photoinduced stiffness-elasticity transition is presented.

## INTRODUCTION

Human skin has the unique features of both soft elasticity and stretchability (*1*). Recently, developing soft elastomer mimicking the human skin has brought about novel applications in wearable electronic devices, flexible electronic packaging, and skin sensors (*2*–*4*). Soft elastomers are a class of cross-linked materials derived with adequately smaller Young’s moduli (ca. 0.1 to 1.0 MPa) than traditional elastomers (ca. 1.0 to 100 MPa) (*5*). This mechanical characteristic can match the Young’s moduli of the human skin (in an order of kilopascal), which allows to fit seamlessly with the human skin and is suitable for wearable applications (*6*). Moreover, soft elastomers are relatively novel bundled materials served as programmable adhesives, which can be used in a wide range of object surfaces (*7*). Several strategies, such as sparse cross-linking polydimethylsiloxane (PDMS), and adding small molecules into chemically cross-linked networks have also been used to prepare soft elastomers (*8*, *9*). While these methods improve elasticity or extensibility of elastomer to a certain extent, they either cause mechanical instabilities or enhance process complexity in the obtained elastomers (*10*). In addition, none of them are universal enough to enable the resulting soft elastomers to be reprocessable and recyclable. Specifically, physical cross-linking has also been explored to prepare supramolecular soft elastomers such as polystyrene-*b*-polybutadiene-*b*-polystyrene (SBS), which is an ABA-type triblock copolymer (TBC) composed of two essential components (*11*–*13*). One is a hard segment with a high glass transition temperature (*T*_g_) above room temperature (RT) serving as a “physical cross-linker” to prevent macroscopic flow, where the physical cross-linker arises from the assembly of glassy polymer upon microphase separation (MPS), and the other is a soft segment with a low *T*_g_ (below RT), which functions as an elastic strand in the network (*14*). This elegantly designed structure provides SBS with the elasticity and the thermoplastic processability (*15*, *16*).

Light is among advantageous, since it can be remotely, instantly, and precisely controlled in one noncontact way (*17*–*22*). Therefore, the photocontrol of mechanical properties of soft materials should have remarkable superiority compared to other stimuli such as temperature, humidity, electrical, and magnetic fields (*23*, *24*). Azobenzene (AZ) is one of the most favorite photoresponsive chromophores due to its reversible trans-to-cis isomerization (*25*–*27*), which has been used to control fluorescence, deformation, and fluidic properties of various materials and even *T*_g_ of AZ-containing polymer (azopolymer) (*28*–*34*). Usually, the trans-rich azopolymer is solid with *T*_g_ above RT, while the cis-rich one are amorphous liquid with *T*_g_ below RT, thus enabling them to undergo a reversible solid-to-liquid transition upon photoirradiation (*35*, *36*). Incorporating AZ moieties into SBS demonstrated both rubber elasticity and photoresponsive properties (*37*–*39*), which could provide novel opportunities to acquire a soft elastomer similar to the human skin.

**Fig. 1.**
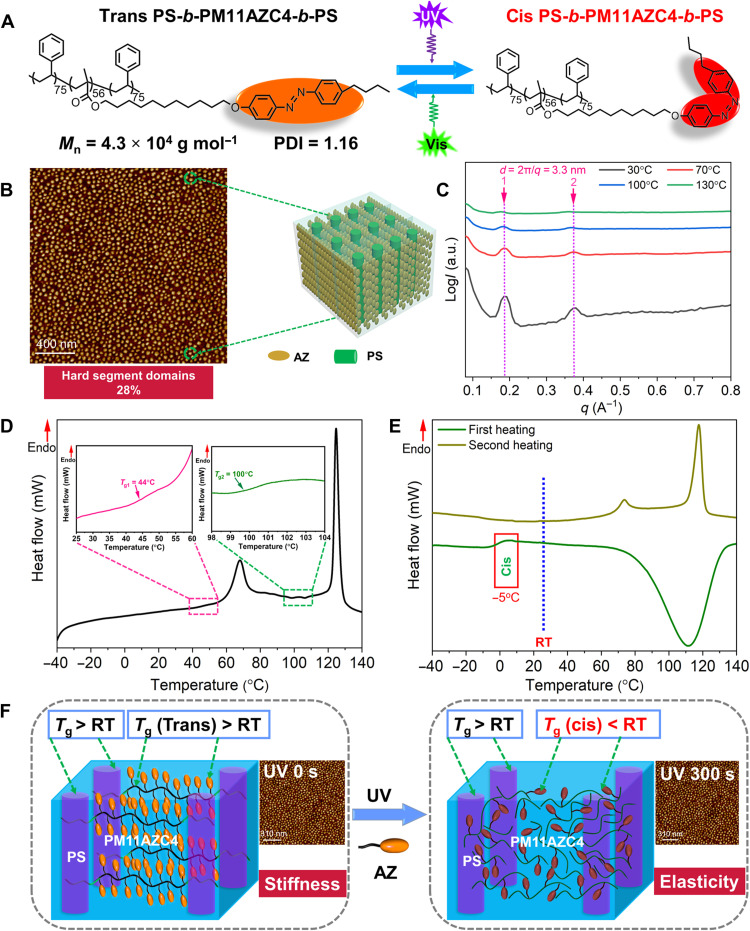
Chemical structure and characterizations of the TBC. (**A**) The chemical structure of TBC, PS_75_-*b*-PM11AZC4_56_-*b*-PS_75_, and its photoisomerization. (**B**) Atomic force microscopy (AFM) phase image of TBC film on silicon wafer. (**C**) SAXS profile for TBC during the heating process with the assignment of the periodic peaks as indicated by closed arrow. a.u., arbitrary units. The heating DSC curves of the trans-rich TBC (**D**) and the cis-rich TBC (**E**) from −40° to 140°C. (**F**) Schematic illustration of the trans- and cis-rich TBC; the inset shows the nanostructures of TBC thin film before and after UV irradiation.

In this work, we design one kind of ABA-type TBC with a well-defined structure by introducing an azopolymer with phototunable *T*_g_ as the middle block and polystyrene (PS) as the end blocks, as shown in Fig. 1A. The midblock (B) of azopolymer (PM11AZC4) is designed as the continuous phase, and the end blocks (A) of PS are designed as the separated phase upon MPS. Both PM11AZC4 and PS show *T*_g_ values higher than RT, and hence the polymer behaves as a stiff plastic at RT. Upon ultraviolet (UV) irradiation, *T*_g_ of PM11AZC4 drops below 0°C, permitting to act as soft domains, while the photoinert PS block serves as “physical cross-links,” making the TBC behave similar to an elastomer, which can be called as “photoinduced elastomer.” This photoinduced mechanically mutable change occurs at RT, and the Young’s modulus of the UV-irradiated TBC is 0.2 MPa, which is a typical soft elastomer mimicking the human skin. To the best of our knowledge, such a convenient approach is the first case to prepare an ideal soft elastomer by way of photo control. The most prominent advantage is that the mechanical mutability between stiffness and elasticity can be athermally achieved in situ without adding any functional small molecules and merely relying on one single polymer material. Furthermore, its nanopattern imprinting on nonplanar substrates especially adaptable for the human skin and RT packaging for perovskite solar cells (PSCs) have been fabricated using the SBS-like TBC, showing that it is promising as advanced soft elastomers for flexible photonic and electronic packaging applications.

## RESULTS

### Design for mechanical mutability

The synthesis and characterization of TBC are given in figs. S1 to S13. The middle block of TBC has a relatively high molecular weight (ca. 2.8 × 10^4^ g mol^−1^) and a narrow polydisperse index (PDI = 1.14; fig. S5) determined by size exclusion chromatography (SEC) with standard PS in tetrahydrofuran so that the middle block has 56 repeat units. Either of the double-ended PS blocks has 75 repeat units, and the obtained TBC with a number-average molecular weight (*M*_n_) of 4.3 × 10^4^ g mol^−1^ and a PDI of 1.16 is marked as PS_75_-*b*-PM11AZC4_56_-*b*-PS_75_. Since the volume fraction of PS in the TBC is calculated as 28%, cylindrical PS microdomains formed in the continuous phase of azopolymer matrix upon MPS, perpendicular to the substrate (Fig. 1B), just as expected. One homopolymer PM11AZC4 with an *M*_n_ of 1.9 × 10^4^ g mol^−1^ and a PDI of 1.12 is used for comparison experiment (fig. S5). As shown in Fig. 1C, the sharp and strong first peak (q*) and the periodic peaks (*q*_peak_) are clearly observed in small-angle x-ray scattering (SAXS) profiles of the TBC, which are assigned to lamellas (*q*_peak_/q* = 1, 2), indicating a lamellar smectic liquid crystal (LC) phase with the interlamellar distance (*d*) of 3.3 nm. While heated up to 130°C, the characteristic peaks disappeared because of the break of ordering at the isotropic phase (Fig. 1C). The pristine TBC shows two *T*_g_ values at 44° and 100°C, respectively, from differential scanning calorimetry (DSC; Fig. 1D). Obviously, the former belongs to PM11AZC4 (*40*), and the latter is attributed to PS blocks. Therefore, segments in the TBC should be restricted by both the rigid PS domains and the frozen LC phase of azopolymer at RT, leading to the pronounced stiffness behavior (movie S1). In accordance with the SAXS results, the DSC curve also confirms that the azopolymer has two transition peaks: the crystal-to-LC transition (*T*_K-LC_) at 68°C and the LC-to-isotropic phase transition (*T*_i_) at 125°C (Fig. 1D).

After UV irradiation, trans-to-cis isomerization of AZs and LC-to-isotropic phase transition occurred simultaneously in the TBC (*35*). Accordingly, the cis-rich azopolymer showed one *T*_g_ of −5°C and a broad exothermic at 110°C due to the thermal cis-to-trans back-isomerization in the first heating curve (Fig. 1E). The photoinduced change in *T*_g_ of the azopolymer may be ascribed to three points: (i) The double bonds in the cis-isomers, which reduce the energy barrier for rotation of adjacent bonds, softening the polymer chain; (ii) the distorted cis-AZ units, which have weaker intermolecular π-π stacking interactions than the conjugated trans-isomers; and (iii) the trans-to-cis photoisomerization of AZs disrupting the LC organization under room light condition (fig. S14). Since *T*_g_ of the azopolymer depends more on entropy than on enthalpy, the LC-to-isotropic phase transition enhances the entropy of system, thereby increasing the free volume of cis-rich azopolymer segments (*40*, *41*). Briefly, these three effects amplify the reduced *T*_g_ of the azopolymer, enabling the TBC to perform as one photoinduced soft elastomer, as shown in Fig. 1F. In addition, no detectable changes in PS nanocylinders of MPS were observed in fig. S15, even the fact that the TBC was irradiated under UV light for 300 s. Because of its light-inert characteristic of the PS block, its photostable *T*_g_ (much higher than RT) should have great influence on mechanical properties of the TBC.

### Contrast in mechanical behaviors and reversibility

**Fig. 2.**
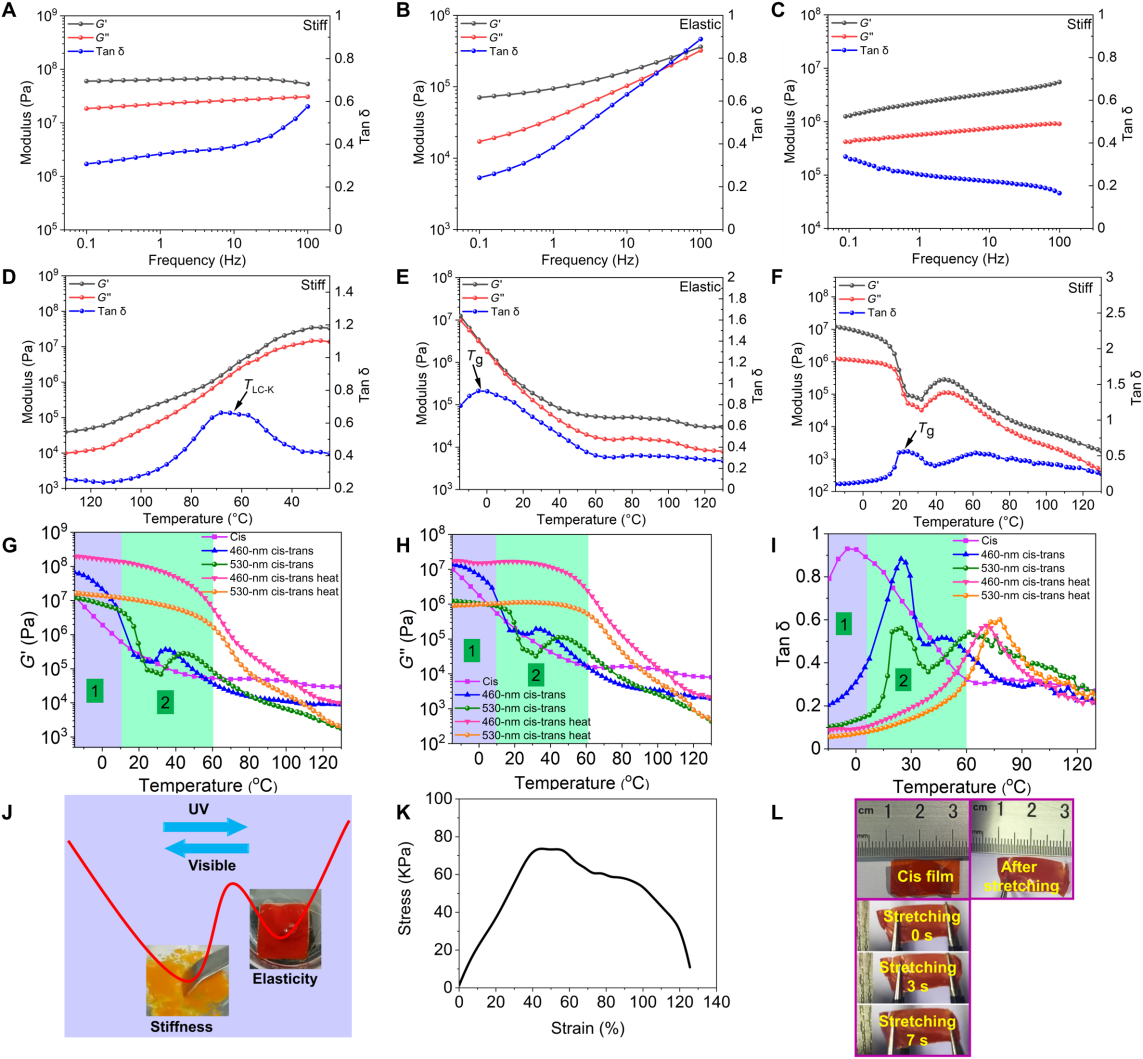
DMA-measured storage modulus (*G*′), loss modulus (*G*″), and loss tangent (tan δ). Frequency sweep of the trans-rich TBC (**A**), the cis-rich TBC (**B**), and the cis-rich TBC after irradiation with 530-nm light for 30 min (**C**) in the whole frequency range (0.01 to 100 Hz) at RT. Temperature sweep of the pristine TBC on cooling from 130° to 25°C (**D**), the cis-rich TBC on heating from −15° to 130°C (**E**), and the restored trans-rich TBC after 530-nm light irradiation on heating from −15° to 130°C (**F**). (**G**) Storage modulus *G*′, (**H**) loss modulus *G*″, and (**I**) loss tangent tan δ as function of temperature for TBC with different treatment: the cis-rich TBC, the restored trans-rich TBC after 460-nm irradiation, the restored trans-rich TBC after 530-nm irradiation, reheating the restored trans-rich TBC after 460-nm irradiation, and reheating the restored trans-rich TBC after 530-nm irradiation, respectively. (**J**) An illustrative plot of potential energy, characterized by an energy barrier between two reversible solid states with distinct mechanical properties. The photographs illustrate the mechanical behavior of the TBC, which is stiff enough in the trans-rich state but soft enough in the cis-rich state. Obviously, it is easy for TBC to achieve the reversible transition between stiffness and elasticity at RT by photo control. Thus, a photoinduced soft elastomer can be obtained by this simple method. (**K**) The stress-strain curve of the cis-rich TBC film. (**L**) The optical images of cis-rich TBC film stretching at different times (movie S2).

Dynamic mechanical analysis (DMA) was used to characterize the phototunable mechanical behaviors of the TBC, and Fig. 2 (A to C) gives the frequency-dependent rheological behavior. As shown in Fig. 2A, its elastic modulus (*G*′) was larger than loss modulus (*G*″), and its loss tangent (tan δ = *G*″/*G*′) was less than 0.60. Both *G*′ and *G*″ remained nearly constant in the whole frequency range (0.01 to 100 Hz) at RT, and the TBC in the glassy state has a *G*′ in an order of nearly 10^8^ Pa. The low tan δ and the high *G*′ demonstrate that the pristine TBC is a rigid and inflexible plastic at RT, and the material tends to fracture under the applied stress, as shown in movie S1. Then, the cis-rich TBC film exhibiting the isotropic phase (fig. S14) was prepared, as shown in fig. S16. Both *G*′ and *G*″ increased with the frequency, but *G*′ was still higher than *G*″ (Fig. 2B). At the low frequency range, *G*″ was proportional to the frequency, and *G*′ remained nearly constant. This is probably because the physical cross-links formed by the PS blocks are almost insensitive and cannot be broken at lower frequencies. Although both *G*′ and *G*″ were greatly reduced by three orders of magnitude comparing to the pristine TBC, the corresponding tan δ was still less than 1.0, unequivocally demonstrating the excellent elasticity.

Then, two different wavelengths of light, 460 nm (fig. S17) and 530 nm (Fig. 2C), were respectively used to induce back-isomerization of the cis-rich sample. Upon exposure for 30 min, *G*′ increased to an order of 10^6^ Pa throughout the frequency range, indicating that visible light irradiation converts cis-AZs back to their trans-forms, consequently increasing the modulus. However, the modulus of the recovered trans-rich TBC was not completely restored to its original value, exhibiting an order of magnitude lower than the pristine sample. This may be ascribed to the thermally reversible nature of the phase transition. When the LC-to-isotropic phase transition is induced by UV light as aforementioned, the reversed phase transition does not occur upon visible light irradiation at RT, since the trans-rich TBC is still in the isotropic phase due to the absent molecular self-organization at highly viscous circumstances (*40*), as shown in fig. S14C. Moreover, the recovery ratio of trans-AZs is lower than that in the initial state, since the photoisomerization is a dynamic equilibrium process (*42*), as shown in fig. S12. Thus, the operation of the phase transition and the molecular isomerization add up to decrease the modulus of the material.

Furthermore, the temperature-dependent rheological behavior of the TBC was also investigated, as shown in Fig. 2 (D to F). On cooling the TBC from 130° to 25°C (Fig. 2D), the isotropic-to-LC phase transition occurred, whereas viscoelastic changes were not detected clearly. As temperature decreased, *G*′ increased gradually, but it was still larger than *G*″, suggesting the appearance of elastic region in which the temperature is very close to the *T*_g_ of PS. Evidently, the modulus changed at one different temperature, corresponding to the LC-to-crystal transition (*T*_LC-K_) of the azopolymer block (Fig. 2D). The TBC became stiff, because *G*′ remained nearly constant below *T*_LC-K_ (RT < *T*_LC-K_). Upon UV light irradiation, the material shows different mechanical behaviors under the same set of environmental condition. As shown in Fig. 2E, a peak of tan δ appears at around −5°C on heating, which can be ascribed to *T*_g_ of the cis-rich azopolymer, in agreement with DSC results (Fig. 1E). At the temperature below *T*_g_ of the cis-rich azopolymer, both enhanced *G*′ and decreased tan δ were observed, indicating the hardening property of glassy polymer. At the temperature higher than *T*_g_ of the cis-rich azopolymer, the transition from glassy to elastic state appeared, as shown in Fig. 2E. *G*′ in the elastic region decreased gradually with elevated temperatures, which could be attributed to a thermosensitive feature of the PS blocks. Since the PS forms nanoscale microphase domains dispersed in the azopolymer matrix, the change in the moduli of PS nanocylinders is not enough to cause an obvious transform of the whole TBC. Hence, the moduli are not greatly changed when the temperature is increased to the *T*_g_ of PS. Conversely, when the temperature reaches the *T*_g_ of the cis-rich azopolymer, the transform in the matrix modulus naturally causes an obvious change in the modulus of TBC.

The reversibility of the phototunable mechanical behavior was studied by exposure to 530-nm light for 30 min, as shown in Fig. 2F. Obviously, the DMA results after visible light irradiation are quite different from the pristine TBC in Fig. 2D. In line with the aforementioned recovery process, irradiation of the cis-rich TBC with 460-nm light manifests the same trend, as shown in Fig. 2 (G to I). Two regions can be distinguished, depending on the temperature conditions. At low temperatures (region 1), *G*′ and *G*″ consequently returned to its original value (Fig. 2, G and H) and eventually reached a modulus plateau. Subsequently, the modulus decreased markedly with increasing temperature (region 2), and a peak of tan δ appeared at around 22°C (Fig. 2I), corresponding to the *T*_g_ of trans-rich azopolymer after visible light irradiation. In this region, the reduced modulus should be exclusively generated by thermal softening. Such a reduction for *T*_g_ of trans-rich azopolymer from 44°C (Fig. 1D) to 22°C is reasonable for the incompletely restored cis-rich azopolymer, which can function as plasticizer. We also compare the modulus of the trans-rich TBC after irradiation with different wavelengths of visible light, and the sample exposed to 530-nm light displays a delayed and slightly weaker stiffening response than that exposed to 460-nm light. On all accounts, upon visible light illumination, the cis-to-trans back-isomerization of AZs were accelerated (half-life period of cis-AZs is 5.6 hours, as shown in fig. S13), and the modulus was increased, further indicating that the mechanical response of the material is reversible at room light condition (Fig. 2J). The molecular-scale integration of the glassy PS domains within an azopolymer matrix allows us to fully leverage the phototunable *T*_g_ and mechanical transition. The photographs in Fig. 2J illustrate the mechanical behavior of the TBC, which is stiff enough in the trans-rich state but soft enough in the cis-rich state.

### Mechanical transition and photoinduced soft elasticity

To further characterize the influence of engineered structure of SBS-like TBC on the mechanical behavior, we compared with that of one homopolymer PM11AZC4. As shown in fig. S18 and tables S1 and S2, the trans-rich homopolymer displayed stiffness of plastic, where *G*′ was in an order of 10^7^ Pa and tan δ of the pristine homopolymer was constantly below 1.0. In contrast, the cis-rich homopolymer exhibited viscous behaviors with tan δ above 1.0, which is far different from the TBC (tan δ <1.0). In addition, a noticeable elasticity was not observed for the cis-rich homopolymer, suggesting that there are no cross-linked networks existing to prevent macroscopic flow. These observations confirm that the mechanical mutability in plastic/elastic behavior is largely dominated by the essential molecular isomerization and physical cross-links of PS microdomain formed via MPS.

Therefore, the mechanical behavior of the UV-irradiated TBC can be defined as an elastic state in a temperature range between *T*_g_ of the cis-rich azopolymer in the continuous phase and *T*_g_ of PS blocks in the separated phase (physical cross-links). As a consequence, this transition can be described as stiff plastic to soft plastic to elastic solid. More precisely, the formation of photoinduced soft elastomer of the TBC includes three elementary steps. The first step corresponds to the trans-to-cis isomerization of AZs under UV light irradiation, and the second one corresponds to the transition from LC to isotropic phase, accompanied with changes from a stiff plastic to a soft plastic state caused by the increased cis-rich azopolymer in the continuous phase. The final step represents the transformation from a soft plastic to an elastic solid via the physical cross-links of glassy PS domains completely surrounded by the maximum ratio of cis-rich azopolymer block with *T*_g_ below RT.

Figure 2K shows the stress-strain curve of the photoinduced soft elastomer of the TBC film. Notably, a distinct yield point was observed, and the storage modulus was estimated as 0.2 MPa, agreeing well with *G*′ evaluated by DMA (Fig. 2E). Figure S19 shows the rate-dependent modulus of cis-rich TBC film, and the soft elastomer’s storage modulus increases obviously with increasing the rate. This is attributed to the time-temperature superposition principle, in which an increase in the rate is shifting the soft elastomer of TBC closer to its glass transition. The cis-rich TBC film exhibited soft elastic, as indicated by stretching at different times of its initial length (Fig. 2L and movies S2 and S3). Therefore, the elasticity of cis-rich TBC could be attributed to the soft middle block in the isotropic phase, which can prevent the local concentration of applied stress in the network and induce energy dissipation during elongation by the segmental mobility, thus rendering the materials soft elastic. Figure S20 displays the elastic stress-strain curves with photocontrollable reversibility. When the soft elastomer of the cis-rich TBC film remaining in the stretched state was exposed in situ to 530-nm visible light, the *G*′ gradually increased, and the elongation at break decreased as exposure time increased (fig. S20 and table S3). Consequently, the soft matter gradually changes from a soft but tough elastomer to a hard and brittle plastic, and no degradation occurs as a result of illumination with light at a low intensity.

The transition between stiffness and elasticity we describe hereafter and above are performed at RT and without the addition of any functional small molecules. Overall, the mechanical properties of the novel SBS-like TBC could be transformed via a mechanism of photoisomerization of AZs, enabling the material to exhibit unique properties of photoinduced soft elastomers, which is far different from SBS or other AZ-containing block copolymer systems (*43*, *44*).

### Programmable mechanics with the static mechanical response

**Fig. 3.**
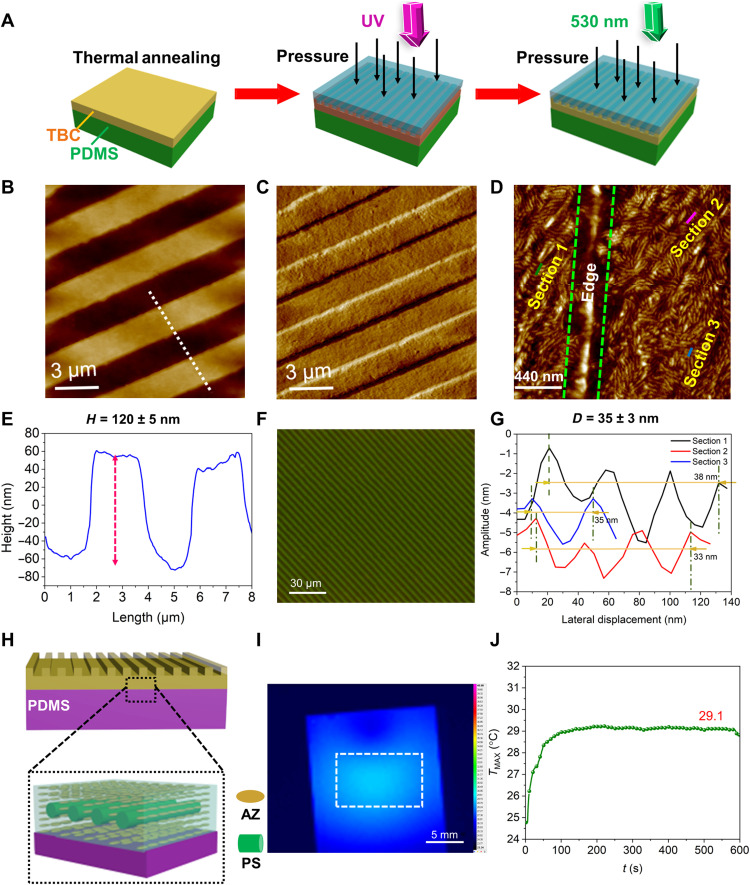
Complex MPS assembling with UV light and exert pressure. (**A**) The nanopattern imprinting includes thermal annealing, imprinting, UV light irradiation, and visible light solidification. (**B**) AFM topography and (**C**) phase images of the imprinted TBC film on PDMS substrate. (**D**) AFM phase image of the imprinted TBC film on PDMS substrate. (**E**) Cross section of the AFM topography image (B) along the indicated white dashed line. (**F**) Optical image of the imprinted TBC film on PDMS substrate. (**G**) The relationship between amplitude and displacement on different sections of AFM phase image (D). (**H**) The combination of external pressure and light irradiation determines the formation of nanosuperstructures formed by MPS of the TBC inside the periodic pattern. (**I**) IR thermal images of the TBC film during UV irradiation, taken at the highest *T*_MAX_. (**J**) *T*_MAX_ as a function of time during 365-nm light irradiation of the TBC film.

To demonstrate the pressure effect on the TBC film, one predesigned PDMS grating mold with a width of 2.0 μm was placed onto the film with an applied mechanical stress (1.35 × 10^5^ Pa) and exposure to UV light (600 s), as shown in Fig. 3A. Thus, the obtained soft elastomer enters the microarray of the mold by deformation. Subsequent visible light illumination (λ = 530 nm, 300 s) induced the reversible elasticity-stiffness transition, leading to a permanent set of new shape (Fig. 3, B and C). A new MPS morphology appeared (Fig. 3D), and atomic force microscopy (AFM) measurement confirms the good replication of the periodic stripes, about 120 nm in depth and 2 μm in width, as shown in Fig. 3E. Optical image in Fig. 3F further indicates the formation of the extensive surface pattern.

For a detailed depiction of Fig. 3D, not only the hardness of substrate but also the action of UV light and exert pressure on film should be considered for the influence on MPS of TBC. Figure S10 shows the morphology of the TBC film on one soft PDMS substrate without the pressure and UV light. Whether the substrate is hard or soft, AFM phase images reveal that the nanocylinder orientation in the lateral plane are perpendicular to the substrate; thus, the hardness of substrate has little effect on of MPS nanostructures. When pressure is exerted on the film surface, the photo-softened azopolymer segments started to deform and moved large scale as time prolongs, which should cause a change in arrangement of PS nanocylinders. Note that the physically cross-linked microdomains of PS were preserved without destruction because of its high *T*_g_, but their arrangement was distorted because of its separated phases upon MPS. Therefore, the combination of external pressure and light irradiation determines the formation of stripe or worm-like MPS, as shown in Fig. 3D. Moreover, the average period of the stripes was obtained as 35 ± 3 nm (Fig. 3G), which is consistent with the intercylinder distance (37 ± 3 nm) obtained from fig. S21. Once the external pressure is applied to the soft elastomer, mass transportation occurs consequently, and the arrangement direction of PS nanocylinders changed from out-of-plane to in-plane arrangement (fig. S22). It is noticeable that there are nanosuperstructures formed upon MPS of the TBC inside the periodic pattern as shown in Fig. 3H, which can extend the patterning abilities of the soft elastomer into multicomponent or three-dimensional structures for versatile patterning.

Comparison experiment was also conducted to show that the photoisomerization is the actual driving force to generate the pattern. When only pressure was directly applied on the pristine TBC film under dark condition without UV light, no any pattern was observed in fig. S23. Then, the pattern can be generated upon UV light irradiation and exert pressure because of photosoftening of the polymer sand formation of soft elastomer. Thus, the elaborately designed structure ensures that the photoinduced soft elastomer does not lose its elasticity in a dynamic environment. Such a behavior can be encoded intrinsically in mechanically responsive materials to mimic not only the static mechanical responses of skins but also the active process they undergo while at work.

To monitor the photothermal effect, the TBC thin film was exposed to 365-nm light, and the temperature was in situ detected by high-resolution infrared (IR) thermography. No noticeable color change was observed from the TBC film (Fig. 3I), and the maximum surface temperature (*T*_MAX_) of the UV-irradiated TBC film reached the highest value of 29.1°C (<*T*_g_ of the trans-rich azopolymer) due to the weak photothermal effect, as shown in Fig. 3J. Thus, the formation of pattern is determined by the action of the isomerization of AZ moieties alone.

### Biomimetic matching of the flexible wearable device

**Fig. 4.**
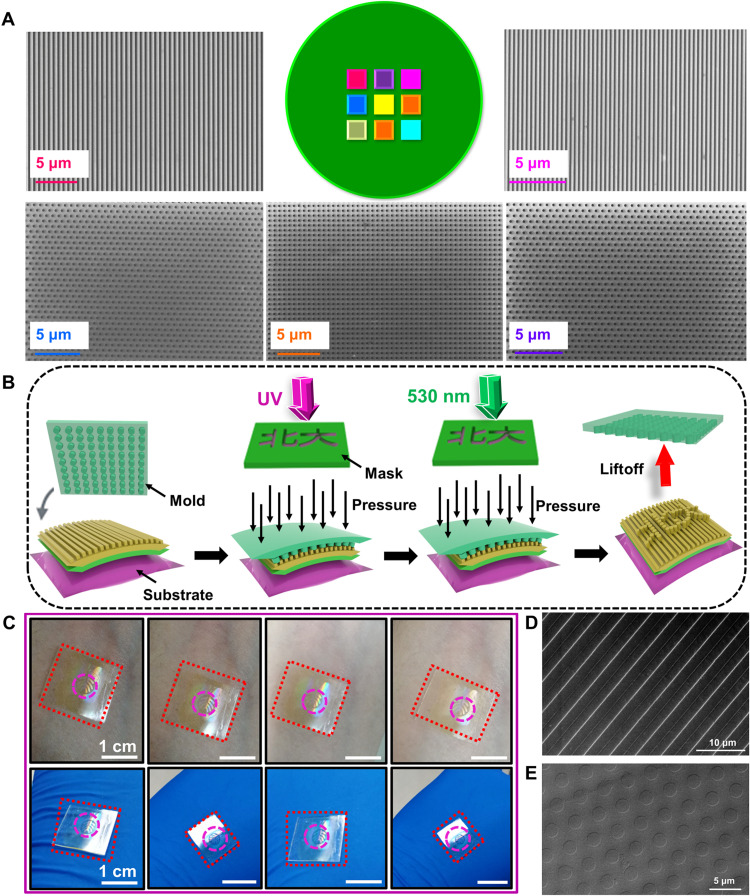
Precise programmable imprinting process via irradiation of the TBC film with different wavelengths of light. (**A**) SEM images taken on different resolution TBC films imprinted with PDMS wafer. Samples were imprinted from five selected locations throughout the wafer; the color code of the scale bar indicates the location on the wafer. (**B**) Formation of a specific pattern: Imprinting with a 3.5-μm hexagonal prism PDMS mold, UV irradiation with a photomask, and visible light solidification. (**C**) The formation of the Chinese characters on the forearm of a person and the back of the hand. (**D**) SEM images of the imprinted TBC film with a 2.0-μm grating PDMS mold. (**E**) SEM images of the secondarily imprinted TBC film with a 3.5-μm hexagonal prism PDMS mold.

On the basis of these results, successful preparation of the soft elastomer with periodic nanostructures inspired us to create different micro-nanopatterns on various types of substrates. Specifically, more than the adaption to planar substrates, the soft elastomer is also applicable to different nonplanar geometries. To demonstrate this feature, we carried out imprinting process via irradiation of the TBC film with different wavelengths of light (fig. S24), where the 3.5-μm hexagonal prism PDMS mold and 2.0-μm grating one were printed onto unconventional substrates such as a convex lens (fig. S25A), a sample bottle (fig. S25B), and the forearm of a person (fig. S25C), respectively. To further increase the spatial resolution, the soft elastomer films were imprinted from five selected locations throughout the PDMS wafer. The PDMS wafer contains nine molds of different resolutions and patterns, and the color code of the scale bar indicates the location of the wafer, as shown in Fig. 4A. Consequently, patterns with a highest resolution of 450 nm can be obtained on the basis of rigid-to-elastic transitions, which is promising for the design of flexible wearable devices. Therefore, the resolution and shape of the nanopattern can be strictly controlled by the mold design.

Many other complex geometries on the human skin can be also generated by means of a sequential process involving external pressure and photoirradiation. The procedure includes, in the first step, fabrication of the patterned substrate with a 2.0-μm grating PDMS mold on the human skin as described in fig. S24. Subsequently, the prepatterned substrate was covered with a 3.5-μm hexagonal prism PDMS mold with the aid of an optical mask, as shown in Fig. 4B. As a simple demonstration, the pattern of Chinese characters was printed on the forearm of a person (Fig. 4C and movie S4) and the back of the hand (Fig. 4C and movie S5). Figure 4D shows the scanning electron microscopy (SEM) images of the imprinted TBC film in the first step. Likewise, Fig. 4E exhibits the good replication of the hexagonal array of pillars after the second imprint process. Obviously, our method combines the advantages of soft elastomer and MPS of TBC, which is very promising at utilization cases for flexible displays in smart phones or computers, wearable technology, and even fabrics.

### Mechanically adaptive adhesion for electronic package

**Fig. 5.**
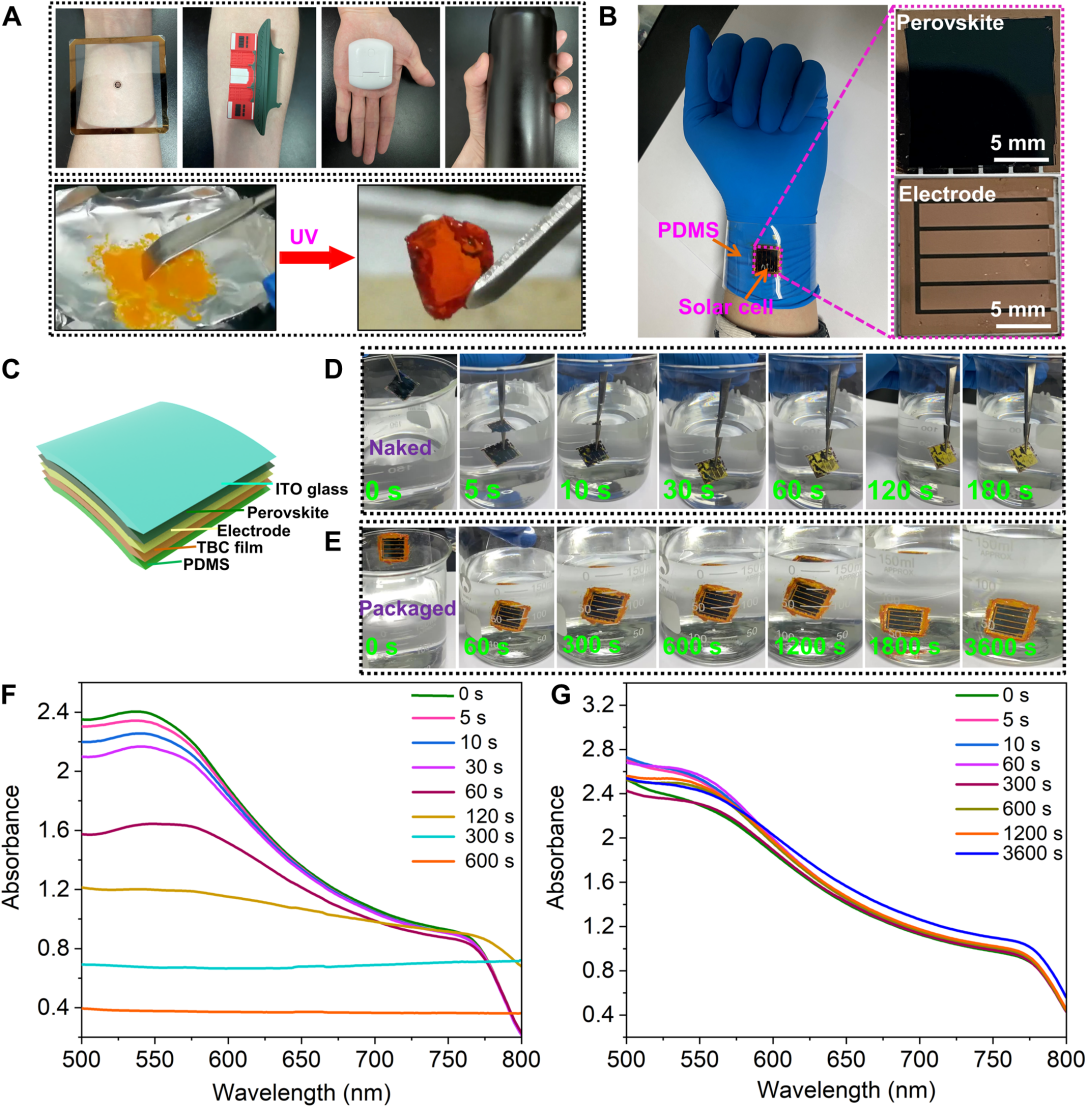
The manufacturing process and characterization of packaged PSC. (**A**) Photographs of the human skin and the biomimetic soft elastomer of TBC film. (**B**) Photographs of packaging PSC and flexible PDMS together using a TBC film. (**C**) Schematic of a layered film structured PSC on flexible PDMS substrate. ITO, indium tin oxide. (**D**) The naked device was completely immerged in water for different times. (**E**) The packaged device via using TBC was immerged in water for different times. UV-visible absorption spectra of (**F**) the naked device and (**G**) the packaged device upon immerging in water recorded at different time intervals, respectively.

Just as expected, this photoinduced soft elastomer can simulate the human skin, which can work on a wide range of surface materials, as shown in Fig. 5A. Therefore, having two reversible solid states with a large mechanical contrast, the obtained elastomer should exhibit stronger capability for seamlessly connecting different shaped objects through elastic deformation, which is very suitable for packaging electronic devices. Compared with traditional encapsulating material, this material has the following advantages: (i) As a consequence of the series of actions at the molecular scale, the photoinduced stiff-to-elastic transition is an athermal process; (ii) this free-standing soft elastomer thin film is suitable not only for current rigid substrate but also for a soft deformable substrate such as PDMS; (iii) the key elements of our strategy are single polymer material, photo, and isomerization. None of these elements will be different. Specifically, the photoinduced softening and stiffening mechanism hinges on a finely controlled molecular process, which is, in principle, not related to solvent; (iv) this material served as the packaging material that can be easily scrubbed off with ethanol, allowing the device to be used repeatedly. As shown in Fig. 5B, we used the TBC film to package one PSC on a flexible PDMS substrate, and obviously, the obtained device can be used for different curved surfaces such as arms. The manufacturing process of this packaged PSC is given in Fig. 5C, and then its water resistance was investigated by immersing in water. As shown in Fig. 5D and movie S6, the naked device began to decompose when immersed in water for 10 s and completely decomposed at 180 s. However, while the packaged PSC was immersed in water for 1 hour, no obvious discoloration was observed (Fig. 5E and movie S6). Subsequently, we quantitatively determined the stability of the naked and packaged devices in water through UV-visible absorption spectroscopy. As shown in Fig. 5F, the absorption intensity of the naked device began to evidently decrease at 30 s and dropped to the minimum value at 600 s. More unexpectedly, even if the packaged PSC was immersed in water for 3600 s, the absorption intensity remained basically unchanged (Fig. 5G), demonstrating that the photoswitchable TBC can efficiently isolate the device from water exhibiting good sealing effect. Therefore, the present soft elastomer is very essential for fabricating electronic devices that works underwater. This unique in situ reversible transformation showing compatibility with easy/scalable fabrication enables our strategy to outperform many existing methods in many areas.

## DISCUSSION

We have demonstrated the athermal mutability of mechanical properties in one novel SBS-like TBC, which has been achieved solely via photoisomerization of AZs. A new concept of photoinduced soft elastomer has been introduced; that is, in response to actinic light, the stiff linear-polymer plastic becomes a noncovalently cross-linked soft elastomer. Consequently, the rationally engineered polymer exhibited unusual mechanical behaviors, reversibly changing between two solid states that can exist under the same set of environment conditions and whose mechanical properties are different by multiple orders of magnitude. The ability to undergo such a large change in mechanical properties highlights the potential of the soft elastomer to create complex nanopatterns on nonplanar substrates especially for the human skin. In addition, the packaged electronic devices have also been manufactured using these soft elastomers that mimics the human skin.

This material could find application as novel mutable materials with a dual nature; furthermore, the design of a new class of photoresponsive block copolymers could be used in photonic devices that not only provide structural support but also can dynamically adjust their properties to the external environment. Although this work has focused on the AZ-containing block copolymer, our approach might be extended to other “two-phase” photoswitchable materials. Integrating photochromic units in linear copolymer to form the one-component system will benefit to design the next generation of responsive materials analogous to some of the complex properties of skins. Nature uses a similar approach on many occasions. This convenient simulation strategy may be of great use in many fields in wearable smart materials, flexible electronic device, self-healing, and photoswitchable adhesive.

## METHODS

### Polymerization

The macromolecular initiators Br-PM11AZC4-Br and TBC, PS_75_-*b*-PM11AZC4_56_-*b*-PS_75_, were synthesized via the atom transfer radical polymerization method. For comparison, we also synthesized the AZ-containing homopolymer PM11AZC4 (*M*_n_: 1.90 × 10^4^ g mol^−1^ and PDI: 1.12). The synthesis and structure of the homopolymer PM11AZC4 and Br-PM11AZC4-Br are completely identical, and only the degree of polymerization is different.

### Nuclear magnetic resonance spectrum

The TBC, PS_75_-*b*-PM11AZC4_56_-*b*-PS_75_, was synthesized by atom transfer radical polymerization of styrene using the macroinitiator Br-PM11AZC4-Br. The chemical shifts for macroinitiator and TBC are assigned in the nuclear magnetic resonance spectra shown in figs. S6 and S7; the resonance signals originating from the phenyl group of azopolymer can be clearly observed at δ = 6.80 to 7.80 parts per million (ppm) (marked as *a*′, *b*′, *c*′, and *d*′ in fig. S7), while those stemming from the phenyl group of PS can be observed at δ = 6.28 to 7.15 ppm (marked as *g*′ and *h*′ in fig. S7B).

### The volume faction of PS block in TBC

The volume fraction of PS block in TBC can be calculated by the following equation, and ρ is the density of polymer block (ρ_PS_ = 1.05 g ml^−1^ and ρ_PM11AZC4_ = 0.80 g ml^−1^). The *M*_n_ values of PS (*M*_PS_) and PM11AZC4 (*M*_PM11AZC4_) are all obtained by SEC measurement

fPS=VPSVPM11AZC4+VPS=MPSρPSMPSρPS+MPM11AZC4ρPM11AZC4=15,0001.0515,0001.05+28,0000.8=0.28

### DSC measurement

To investigate the *T*_g_ of PS_75_-*b*-PM11AZC4_56_-*b*-PS_75_ after UV irradiation, we used a “solution method” to prepare the cis-rich TBC for DSC measurements. First, the 10.0 weight % (wt %) TBC solution in dichloromethane (DCM) was irradiated with UV light at 365 nm for 30 min. Then, the irradiated TBC solution was drop-coated on the clean glass. The film was placed at RT to completely remove the solvent while maintaining the UV light at the same time to prevent the cis-to-trans isomerization. Last, the UV-irradiated TBC was studied via DSC. DSC measurements were performed with DSC 8000 (PerkinElmer, Boston, USA).

### AFM measurement

We used AFM to characterize the morphology feature of the TBC. TBC film was thermally annealed at 130°C for 24 hours. In fig. S10, regarding PS_75_-*b*-PM11AZC4_56_-*b*-PS_75_, for which *f*_PS_ = 0.28, the packed cylinders of the nanophase structures are formed. AFM phase images revealed that the cylinder orientation in the lateral plane was perpendicular to the substrate.

### Preparation of the cis-rich TBC film

A 1.0 wt % sodium PS sulfonate solution in water was first spin-coated onto a clean glass substrate. The 10.0 wt % TBC solution in DCM was irradiated with a UV light at 365 nm for 30 min. Then, the irradiated TBC solution was drop-coated on the anchored substrate layer. Last, the bilayer film was immersed in water to obtain the freestanding TBC film, as shown in fig. S16.

### DMA measurement

DMA was conducted on an Anton Paar MCR 301 rheometer. Oscillation mode was applied under deformation-controlled condition. Parallel plate-measuring system was used with a diameter of 8 mm. Before the rheological tests, we need to determine the linear viscoelastic range. A γ of 3% was chosen for the cis-rich TBC and 0.1% for trans-rich one in the rheological test. For the temperature tests, the storage and loss moduli of cis-rich TBC were recorded during heating from −15° to 130°C. For trans-rich TBCs, they were recorded during cooling from 130° to −15°C. Both the cooling rate and the heating rate are 3°C per min. One hertz was set as the frequency.

### The fabrication of PSC

The fabrication of PSC is based on the previous report (*45*).

## Supplementary Materials

### This PDF file includes:

Supplementary Text Figs. S1 to S25 Tables S1 to S3 References
