# Supplementary material for: Mechanically mutable polymer enabled by light
Source: Sci Adv. 2022 Aug 24;8(34):eabo1626. doi: 10.1126/sciadv.abo1626 (PMC9401616; doi:10.1126/sciadv.abo1626)
Supplement: Supplementary file 1 — Supplementary Text Figs. S1 to S25 Tables S1 to S3 References [file sciadv.abo1626_sm.pdf]

Science Advances
AAAS

Supplementary Materials for

Mechanically mutable polymer enabled by light

Feng Cai *et al.*

Corresponding author: Haifeng Yu, yuhaifeng@pku.edu.cn; Wei Feng, weifeng@tju.edu.cn

Sci. Adv. **8**, eabo1626 (2022)
DOI: 10.1126/sciadv.abo1626

The PDF file includes:

Supplementary Text
Figs. S1 to S25
Tables S1 to S3
Legends for movies S1 to S6
References

Other Supplementary Material for this manuscript includes the following:

Movies S1 to S6

Materials

Tetrahydrofuran (THF; Beijing Chemical Plant, Beijing, China; 99%) was distilled after refluxing over anhydrous calcium hydride (CaH_2; Tianjin Jinke Fine Chemical Industry, Tianjin, China; 97%). Styrene (St; Aladdin; 99%) was dried over anhydrous calcium hydride and then distilled under vacuum. Copper(I) bromide (Cu(I)Br; Alfa, Lancashire, United Kingdom; 98%) was washed with acetic acid and ethanol three times, and then dried under vacuum. Butylaniline (Acros, Geel, Belgium; 99%), phenol (Sinopharm Chemical Reagent, Shanghai, China; 99%), 11-bromo-1-undecanol (Adamas; 98%), hydrochloric acid (Xilong Science, Shantou, China; 99%), methacryloyl chloride (Alfa; 97%), hexamethyltriethylenetetramine (HMTETA; TCI; 98%), pentamethyldiethylenetriamine (PMDETA; 99%), potassium iodide (KI; Sinopharm Chemical Reagent; 99%), chloroform-d for ^{1}H NMR ($CDCl_3$; Beijing Innochem Technology, Beijing, China; 99.8%), dichloromethane-d2 for ^{1}H NMR (CD_2Cl_2; Beijing Innochem Technology, Beijing, China; 99.8%), dichloromethane (DCM; Beijing Tongguang Fine Chemical Industry; 99%), ethanol (Beijing Tongguang Fine Chemical Industry; 99%), and methanol (Beijing Tongguang Fine Chemical Industry; 99%) were used as received. The water-soluble ionic polymer, sodium polystyrenesulfonate (PSSNa) with weight-average molecular weight of 7×10^4 g mol^{-1} was obtained from Aldrich and used as received.

Instruments and measurements

^{1}H-NMR spectrum was recorded at room temperature (RT) on Bruker AVANCE III HD 500M (NMR; Bruker; Germany) using deuterated chloroform ($CDCl_3$) or deuterated dichloromethane (CD_2Cl_2) as the solvent with tetramethylsilane (TMS) as an internal standard. The molecular weights of polymers were determined by size exclusion chromatography (SEC; Waters; USA) with standard polystyrenes (PSs) in THF as eluent. UV/Vis absorption spectra were recorded on a Lambda 750 spectrophotometer (PerkinElmer; Boston, USA). Differential scanning calorimetry (DSC) measurements were carried out with DSC 8000 (PerkinElmer; Boston, USA) at the heating rate of 10 °C min^{-1} under nitrogen. Polarized optical microscopy (POM) was performed on a Scoper A1 polarizing optical microscope (Zeiss; Germany) equipped with a hot stage. The microscopic topography of films was evaluated with an atomic force microscope (AFM; Bruker Multimode 8 in a tapping mode; Germany). Small-angle X-ray scattering (SAXS) experiments were performed with Ganesha system (SAXSLAB; Anton Paar; USA) equipped with a multilayer focused Cu Kα radiation as the X-ray source. The wavelength of the X-ray beam was 1.54 Å. The scattering vector q ($q = 4\pi\sin\theta/\lambda$, where λ is the X-ray wavelength and 2θ is the scattering angle) was calibrated using copper behenate. Dynamic mechanical analysis (DMA) was conducted on an Anton Paar Physica MCR 301 rheometer using an 8 mm parallel plate geometry. A gap spacing of ~0.2 mm was used for all measurements. Dynamic shear moduli (G' and G'') were examined in the linear viscoelastic regime. The films were stretched using a material universal testing machine (CMT-10; Jinan Liangong Testing Technology; China). The surface microstructures of thin films were obtained by scanning electron microscopy (SEM; HITACHI, S-4800; Japan). The surface temperatures of the photoirradiated films were simultaneously monitored by an infrared camera (FLIR SC7000).

Polymerization

The monomer M11AZC4 and the initiator (Fig. S1) were synthesized according to previous reports.(*46,38*) The ^{1}H-NMR data of the monomer M11AZC4 is shown in Fig. S4. The triblock copolymer (TBC) PS-*b*-PM11AZC4-*b*-PS was synthesized via the atom transfer radical polymerization (ATRP) method.

Synthesis of 1,1'-biphenyl-4,4'-bis(2-bromoisobutyrate) (Dibromo Initiator)

4,4'-biphenol (0.93 g, 5.00 mmol), triethylamine (1.11 g, 11.00 mmol), and THF (40 mL) were added in a 100 mL flask. Bromoisobutyryl bromide (2.53 g, 11.00 mmol) was added dropwise under stirring. The reaction was continued at RT overnight. Afterward, the mixture was poured into water, and a white solid precipitated. After filtration and recrystallization in ethanol, the white pallet crystal was obtained. ^{1}H NMR (500 MHz, $CDCl_3$) δ: 2.1 (s, 12H), 7.20 (d, 4H), 7.6 (d, 4H), as shown in Fig. S3.

Synthesis of Br-PM11AZC4-Br macroinitiator by ATRP

The dibromo initiator, 1,1'-biphenyl-4,4'-bis(2-bromoisobutyrate) (39.2 mg, 0.081 mmol), M11AZC4 (3.19 g, 6.48 mmol) were placed in a 50-mL Schlenk flask. In a glove box, CuBr (11.6 mg, 0.081 mmol), HMTETA (18.7 mg, 0.081 mmol) and the dry anisole (5 mL) were placed in the 50-mL Schlenk flask under an N_2 atmosphere. Then the sealed flask was removed from the glove box and stirred for 30 min at RT. The reaction was carried out by immersing the flask in a preheated oil bath at 85 °C. After polymerization for 24 h, the flask was immersed in liquid nitrogen. The reaction mixture was diluted with DCM (20 mL), passed through a column of alkaline alumina to remove the Cu(I) catalyst, and the product was precipitated by pouring the mixture into methanol (200 mL). It was collected by filtration and pouring into methanol. The abovementioned process is repeated three times and the obtained polymer was dried in vacuo at ambient temperature for 24 h to afford Br-PM11AZC4-Br as a yellow powder.

Synthesis of PS-*b*-PM11AZC4-*b*-PS by ATRP

The Br-PM11AZC4-Br macroinitiator (1.00 g, 0.036 mmol) was placed in a 50-mL Schlenk flask. In a glove box, CuBr (15.5 mg, 0.108 mmol), St (1.12 g, 10.8 mmol), PMDETA (18.7 mg, 0.108 mmol) and the dry anisole (1 mL) were placed in the 50-mL Schlenk flask under an N_2 atmosphere. Then the sealed flask was removed from the glove box and stirred for 30 min at RT. The reaction was carried out by immersing the flask in a preheated oil bath at 90 °C. After polymerization for 24 h, the flask was immersed in liquid nitrogen. The reaction mixture was diluted with DCM (20 mL), passed through a column of alkaline alumina to remove the Cu(I) catalyst. The product was precipitated by pouring the mixture into hot methanol (400 mL) and the yellow solid polymer power was collected by filtration. And the process is repeated three times. Finally, the polymer was dried in vacuo at ambient temperature for 24 h to afford PS-*b*-PM11AZC4-*b*-PS as a yellow powder. And the polymerization process is shown in the Fig. S2.

SEC measurement

Synthesis of Br-PM11AZC4-Br macroinitiator

The Br-PM11AZC4-Br macroinitiator with 56 repeat units was first prepared via ATRP. The SEC trace for the macroinitiator after reprecipitation is shown in Fig. S5.

Synthesis of TBC, PS-*b*-PM11AZC4-*b*-PS

The Br-PM11AZC4-Br was then used as a macroinitiator to synthesize azobenzene (AZ)-containing TBC in a typical ATRP reaction. The growth process was confirmed by SEC, which showed a continuous decrease in elution time (Fig. S5), indicating a controlled

increase in molecular weight upon chain extension from the macroinitiator Br-PM11AZC4-Br to the TBC.

The liquid crystal (LC) properties of the TBC

The LC properties of the TBC were investigated using DSC and POM. Endothermal peaks due to phase transitions at the crystal-to-LC transition ($T_{K\text{-}LC}$) and LC-to-isotropic phase transition (T_i) are evident in the DSC curves (Fig. 1D); these phase transitions are also reversible.

POM was used to observe the texture of the TBC. Solid samples were first heated to 180 °C and then cooled to RT. Images were captured during the cooling process. The image of the Br-PM11AZC-Br at 35 °C is shown in Fig. S8. And the POM images of the PS_{75}-*b*-$PM11AZC4_{56}$-*b*-PS_{75} between 109 °C and 35 °C are shown in Fig. S9.

UV-Vis absorption spectra

The photoresponsive behaviors of the TBC were studied in film upon irradiation of UV light (365 nm, 100 mW/cm^2, Fig. S12A) and then visible light (530 nm, 50 mW/cm^2, Fig. S12B). As shown in Fig. S12A, the spectrum initially showed an intense π-π* band in the UV region and a weak n-π* band in the visible region, indicating the existence of abundant trans AZs. The maximum absorption in film blue-shifted from 359 to 338 nm compared to that in solution in Fig. S11, attributed to the formation of H-aggregation and the parallel stacking of AZ mesogens.(*47*) Upon UV irradiation (365 nm) from 0 to 1.5 s, the π-π* band intensity decreased whereas the n-π* band intensity increased concomitantly, suggesting the occurrence of trans-to-cis isomerization of AZs (Fig. S12A). Subsequent green light irradiation (530 nm) from 0 to 46 s induced the opposite cis-to-trans isomerization of AZs (Fig. S12B). The half-life period of cis isomer was measured as 5.6 h (Fig. S13), much longer than electron push-pull AZs.(*48*)

The stability of microphase separation (MPS) nanostructures

The MPS domains of PS blocks in TBC was designed as the physical cross-links supporting the extension of the continuous phase of PM11AZC4, which should play an important role in achieving the conversion between elasticity and stiffness. Since PS is photo-inert, we speculate that whether the UV irradiation could change MPS morphologies of the TBC. As previously reported, the regularly patterned cylindrical nanostructures in poly (ethylene oxide) (PEO)-based AZ-containing LC block copolymers could be quickly photo-directed from in-plane to out-of-plane arrangement at RT.(*49*) The present TBC showed nanocylinders of PS domains perpendicularly to substrate upon thermal annealing, as shown in Fig. S15A. However, no detectable changes in MPS nanostructures were observed in Figs. S15B-S15D even upon irradiation of UV light for a long time (300 s). At this case, AZs of azopolymer block should be in the cis-rich photostationary state, in which the stiff PS domains was dispersed, serving as the physical cross-links. As a result, the possible change in MPS nanostructure of the TBC can be given in Fig. S15E. Both phase transition from LC to isotropic and decreased T_g were induced in the AZ-containing block upon UV irradiation, but the PS nanocylinders formed by MPS was not influenced due to its light-inert characteristic and its T_g higher than RT, which should have great influence on mechanical properties of the TBC.

The nanopattern imprinting using TBC film

Here, the TBC dissolved in THF was spin-coated on a poly (dimethylsiloxane) (PDMS) substrate to prepare a uniform thin film, which was thermally annealed at 130 °C for 13

h. By the way, PDMS was chosen as the substrate because its flexible and transparent properties, allowing for successful attachment and bending of the TBC. Thus, it can be engineered for desired applications. Then the pre-designed PDMS grating mold with the periodicity of 2 μm was placed onto the film with an external mechanical stress (1.35 × 10^5 Pa) and UV light (600 s) applied. Subsequently, 530 nm visible light is used for photocuring, as shown in Fig. S24.

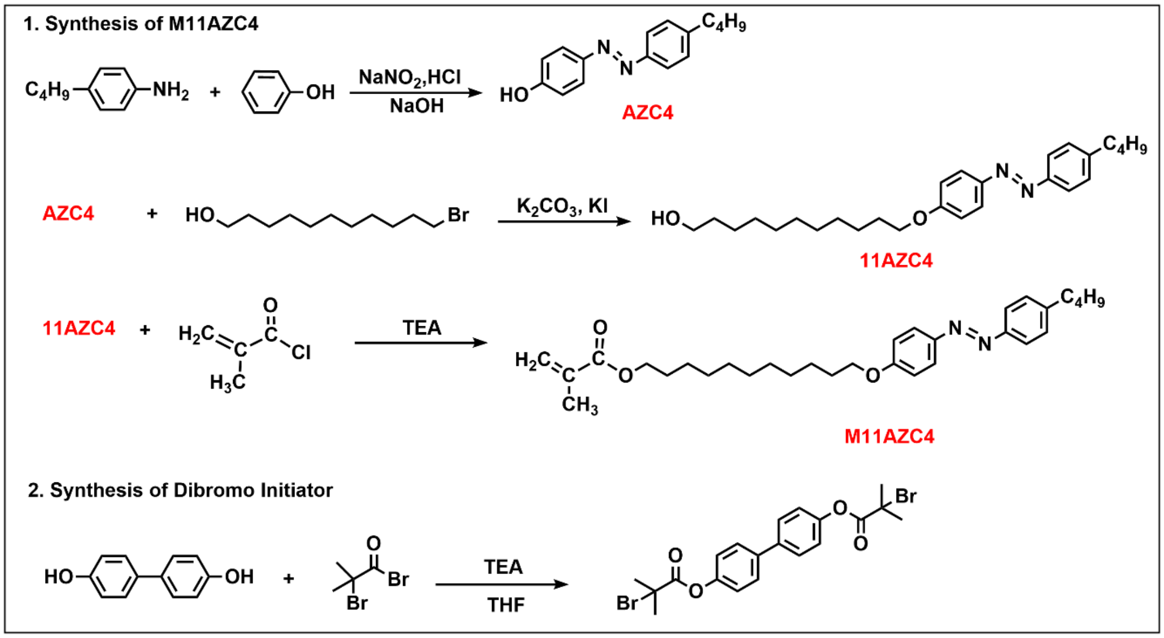

Fig. S1.
The Synthesis route of the monomer M11AZC4 and the dibromo initiator.

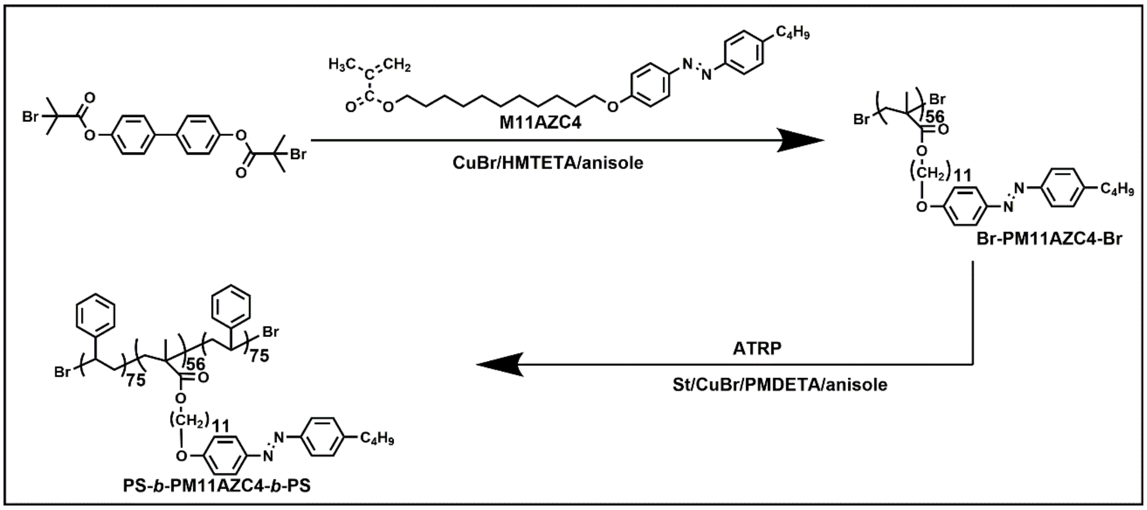

Fig. S2.
Synthesis route of the TBC, PS_{75}-*b*-$PM11AZC4_{56}$-*b*-PS_{75}.

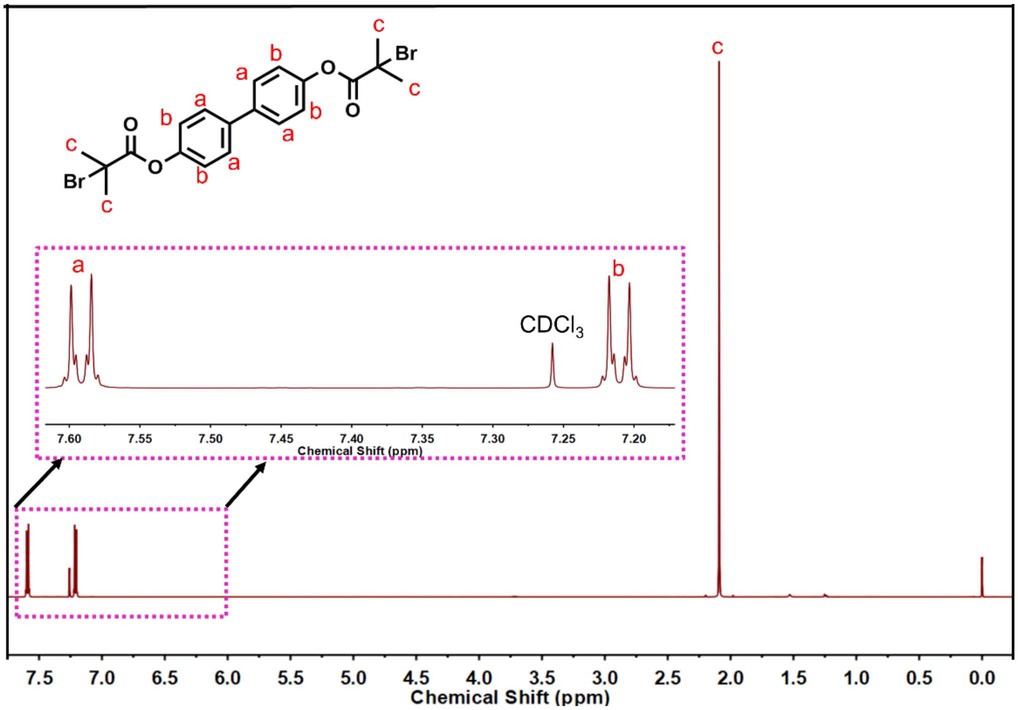

Fig. S3.
[1]**H NMR spectrum** of the dibromo initiator in $CDCl_3$ at room temperature.

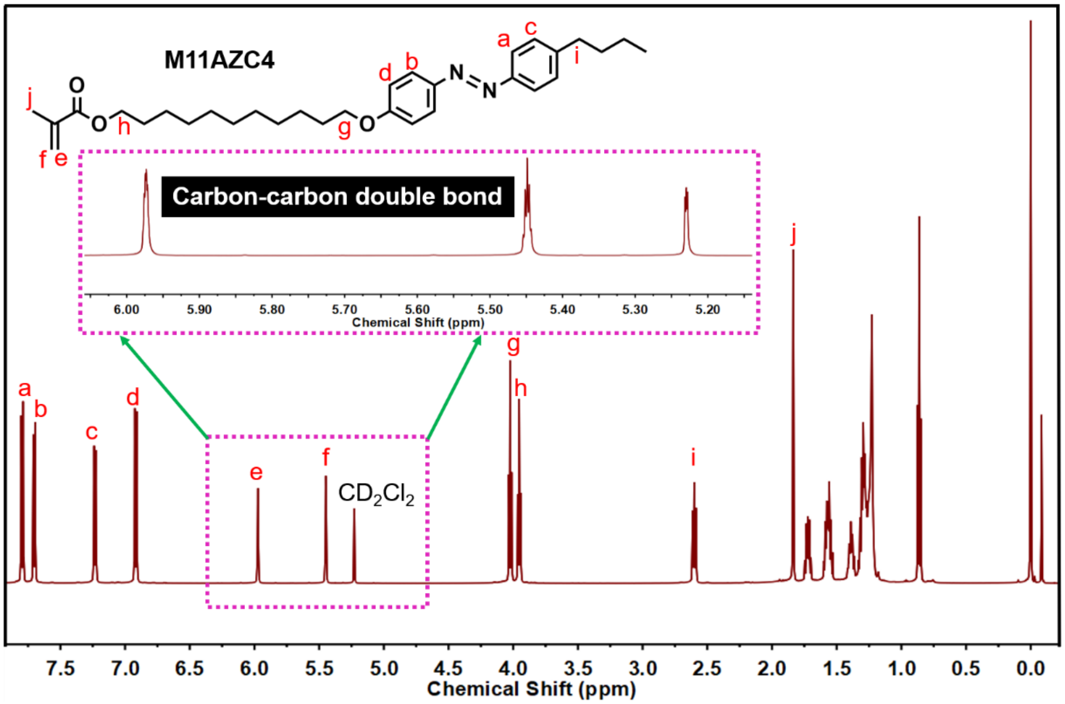

Fig. S4.
[1]**H NMR spectrum** (CD_2Cl_2; room temperature) of the monomer M11AZC4.

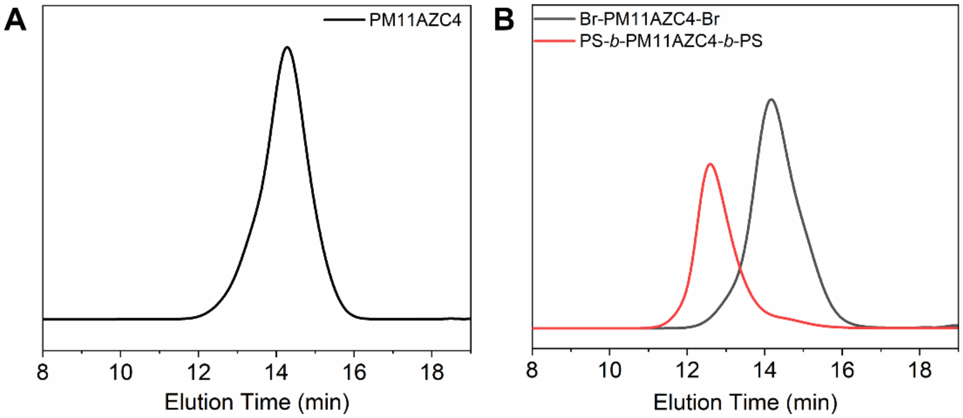

Fig. S5.

SEC traces for (A) the homopolymer PM11AZC4 and (B) the Br-PM11AZC4-Br macroinitiator and TBC, PS_{75}-*b*-$PM11AZC4_{56}$-*b*-PS_{75}.

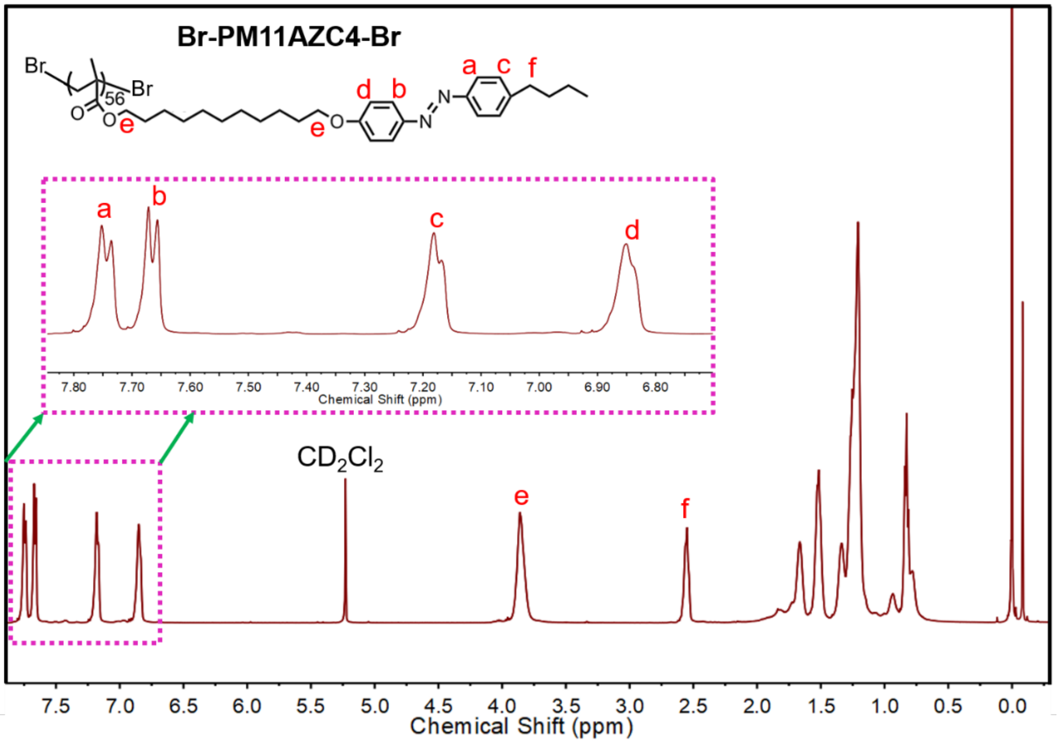

Fig. S6.

^{1}H NMR spectrum (CD_2Cl_2; room temperature) of the macroinitiator Br-PM11AZC4-Br. The inset shows selected region of ^{1}H NMR spectrum of Br-PM11AZC4-Br.

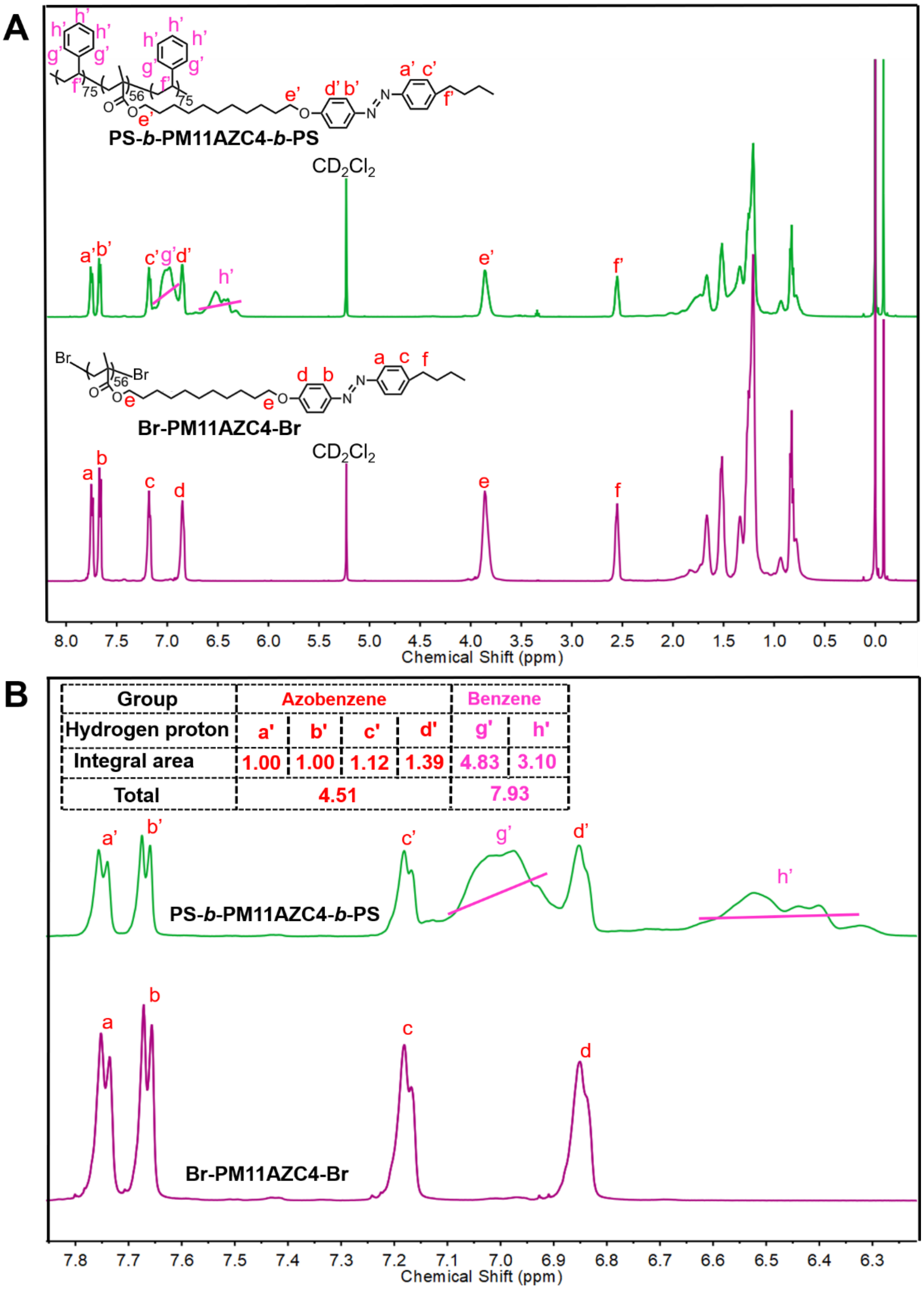

Fig. S7.
(A) ^{1}H NMR spectra (CD_2Cl_2; room temperature) of the TBC (top), PS_{75}-*b*-$PM11AZC4_{56}$-*b*-PS_{75} and the macroinitiator Br-PM11AZC4-Br (bottom). (B) Selected region of the ^{1}H NMR spectra of the TBC (top) and the macroinitiator Br-PM11AZC4-Br (bottom).

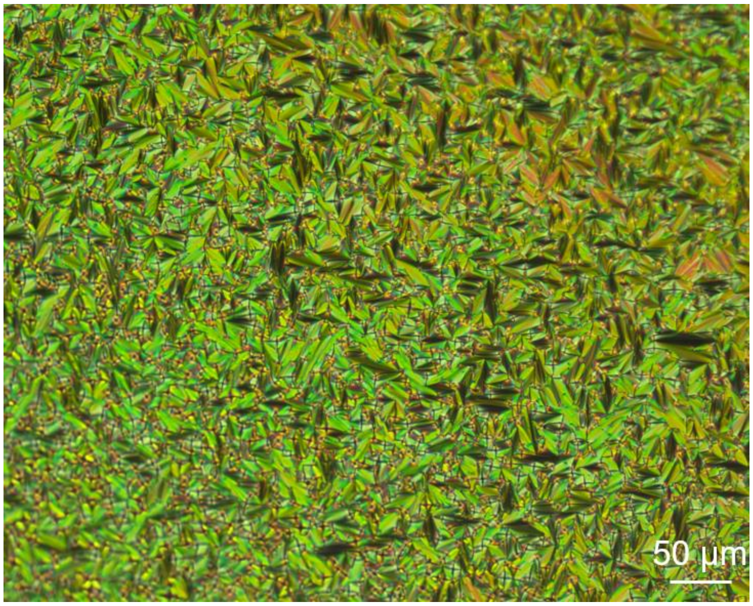

Fig. S8.
POM image of the macroinitiator Br-PM11AZC4-Br at 35 °C.

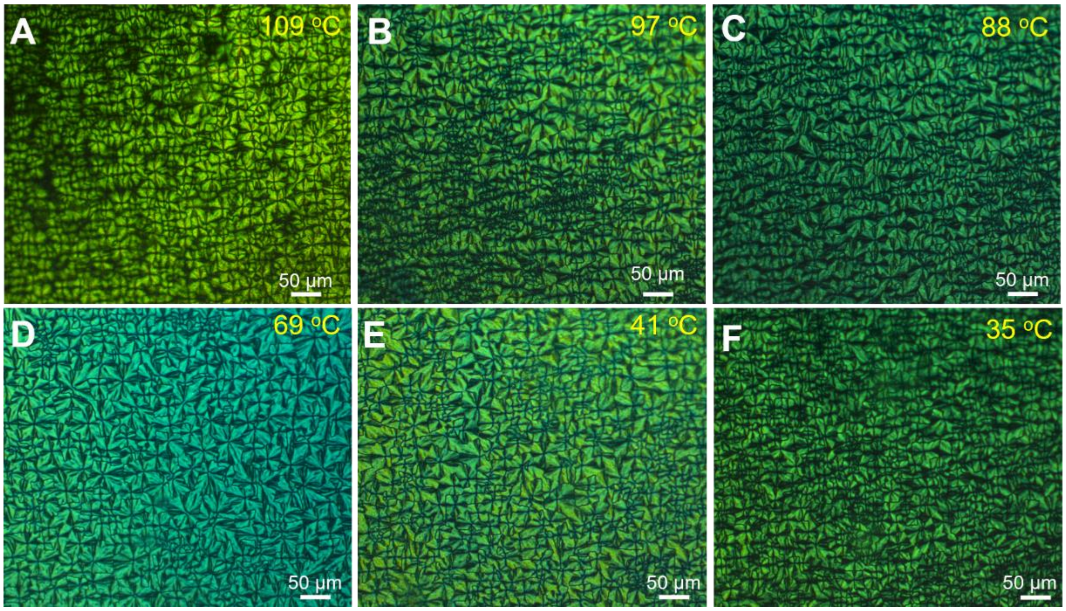

Fig. S9.
POM images of the TBC at different temperatures: (A) 109 °C; (B) 97 °C; (C) 88 °C; (D) 69 °C; (E) 41 °C; (F) 35 °C.

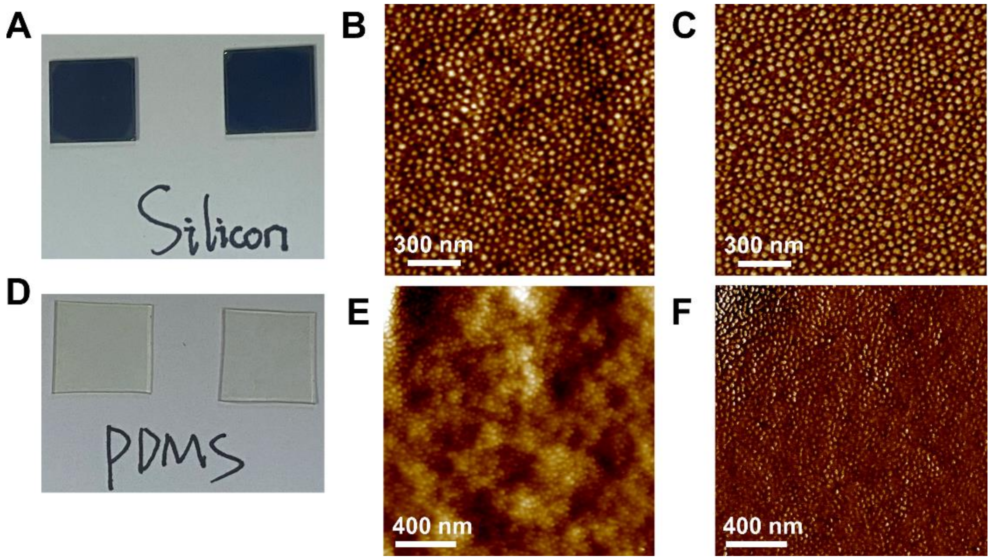

Fig. S10.

The MPS results of TBC film. (A) The image of the TBC film spin-coated on silicon surface. (B) AFM height and (C) phase images of TBC film on silicon after thermal annealing. (D) The image of the TBC film spin-coated on PDMS surface. (E) AFM height and (F) phase images of TBC film on PDMS after thermal annealing.

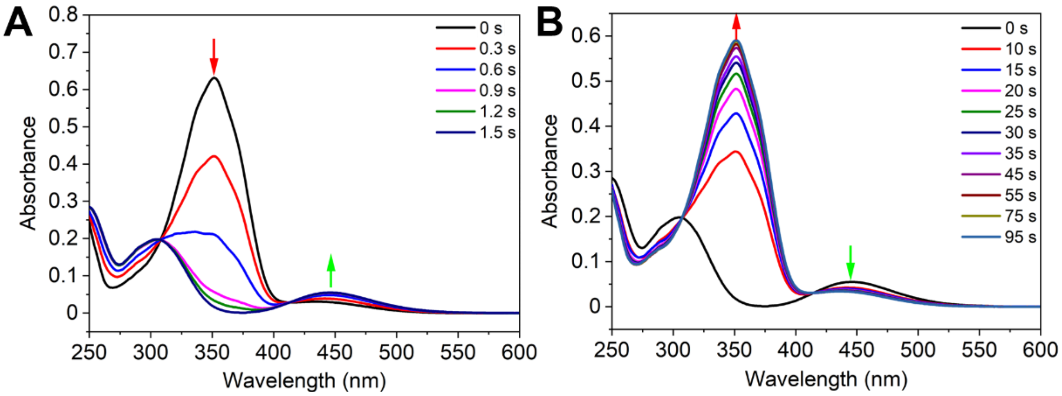

Fig. S11.

UV-Vis absorption changes of TBC solution in THF upon photo irradiation with (A) UV light at 100 mW cm^{-2} and (B) 530 nm light at 50 mW cm^{-2}.

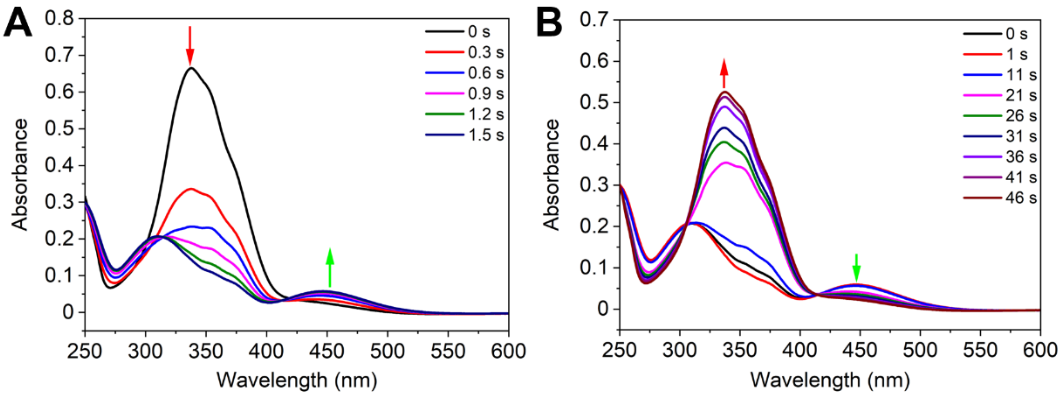

Fig. S12.

UV-Vis absorption changes of TBC thin films upon photo irradiation with (A) UV light at 100 mW cm^{-2} and (B) 530 nm light at 50 mW cm^{-2}.

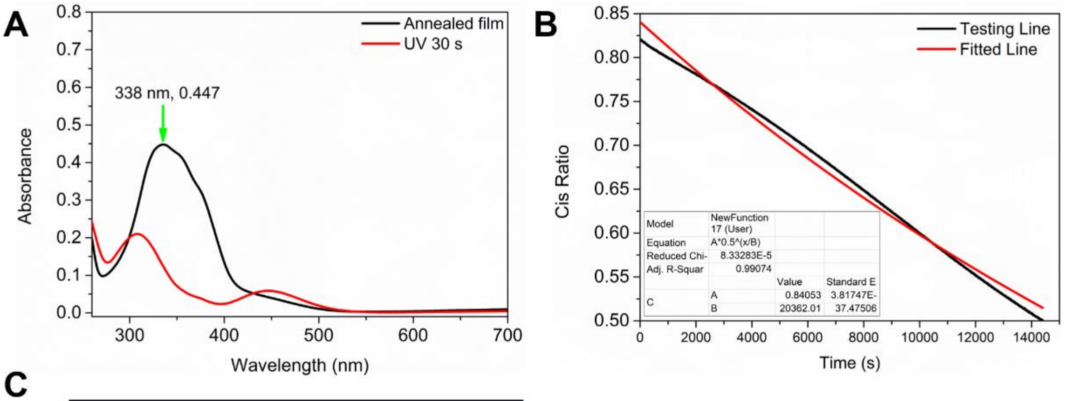

C

Half-life Equation	y=A*0.5^(x/B)
Fitted A	0.84053
Fitted B	20362 s/5.6 h
Adjusted R-square	0.99074

Fig. S13.

Half-life curve fitted by the data obtained from UV-Vis absorption spectra of TBC film on quartz plate in a dark environment. (A) UV-Vis absorption changes of TBC film upon irradiation with 365 nm UV light at 100 mW cm^{-2}. (B) First-order kinetic plots of data for the cis-to-trans thermal isomerization of AZ groups in film at different time. The black line is the actual test result, and the red line is the fitted one. The inset presents the fitting formula and parameters we used. (C) Half-life curve fitted by the data obtained from UV-Vis absorption spectra of TBC film on quartz plate in a dark environment. The sample was first irradiated with enough 365 nm light illumination and then immediately tested on its absorbance at 338 nm.

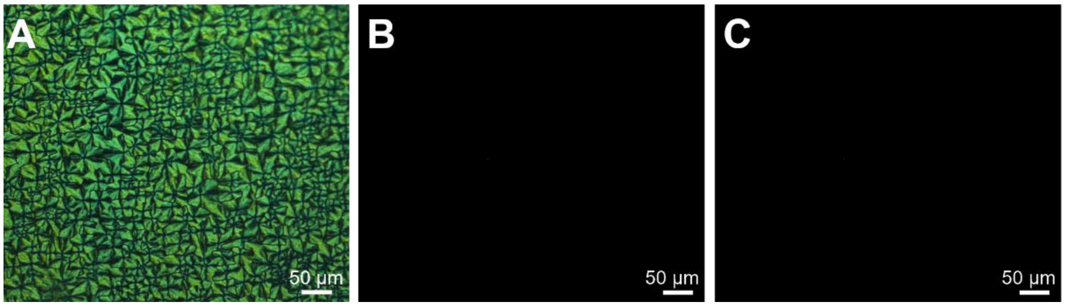

Fig. S14.
POM images of TBC film (A) after thermal annealing, (B) upon irradiation with 365 nm UV light at RT, (C) upon irradiation with 530 nm light at RT.

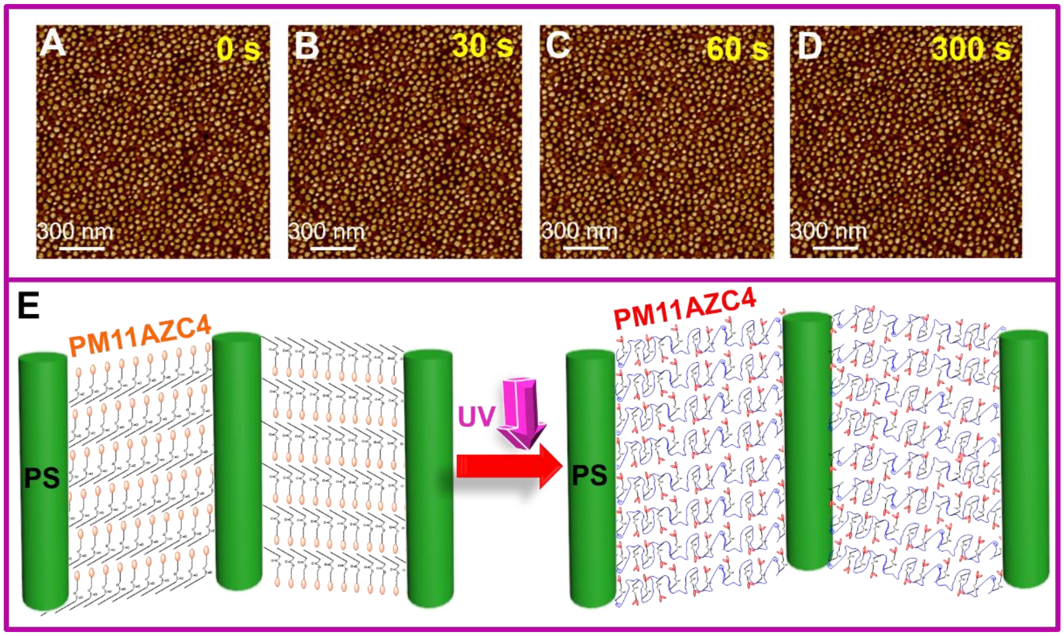

Fig. S15.
AFM phase images and schematic illustration of the MPS nanostructures. AFM phase images (A), (B), (C) and (D) of TBC film irradiated with UV for 0 s, 30 s, 60 s and 300 s, respectively. (E) Schematic illustration of MPS structure and photoinduced phase transition of the TBC film.

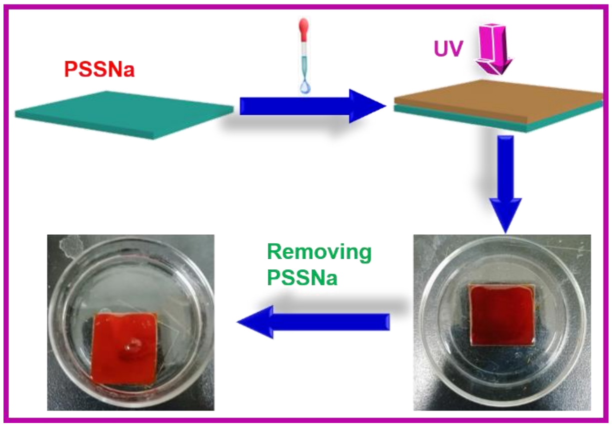

Fig. S16.
The preparation process of the cis-rich TBC film.

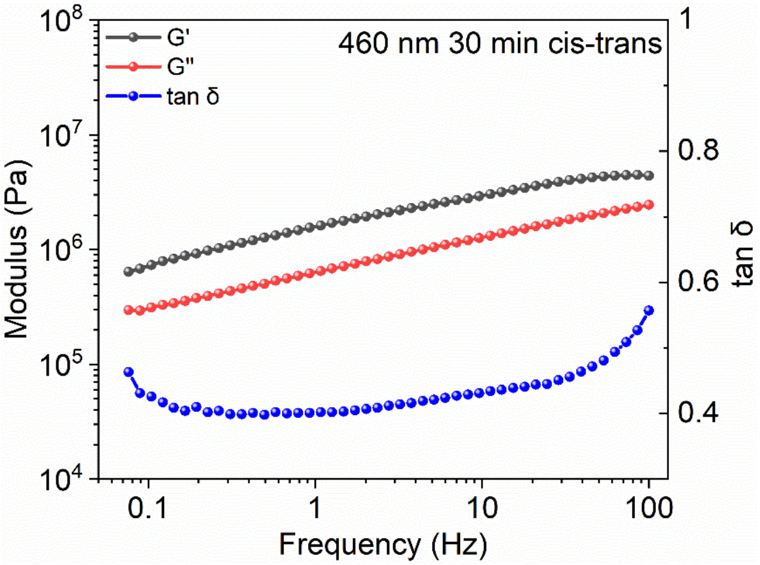

Fig. S17.
DMA-measured storage modulus (G'), loss modulus (G'') and loss tangent (tan δ).
Frequency sweep of cis-rich TBC after being irradiated with 460 nm light for 30 min.

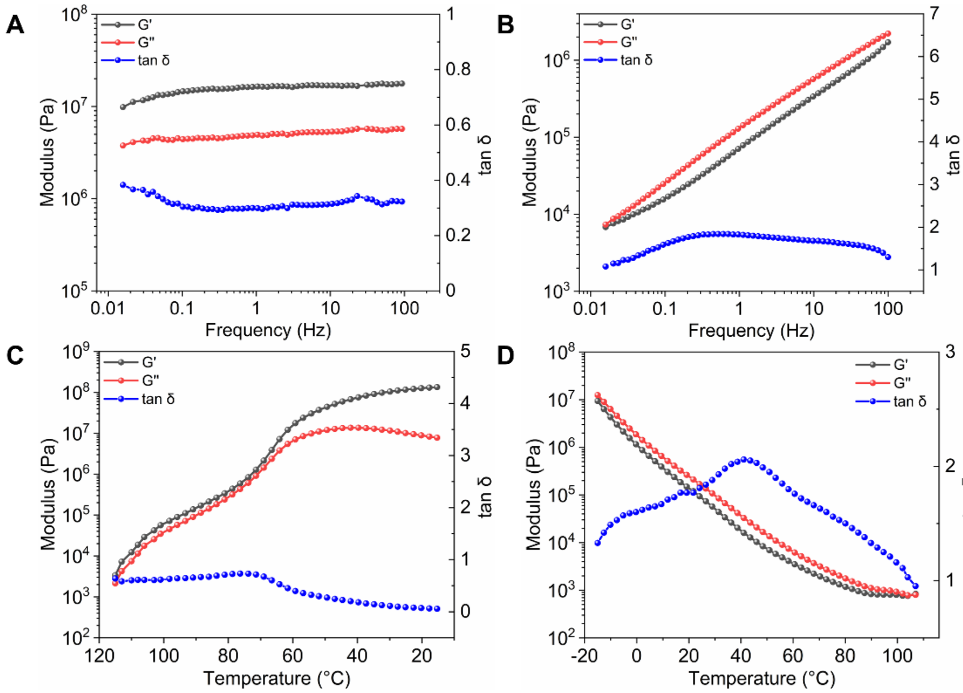

Fig. S18.
DMA-measured storage modulus (G'), loss modulus (G'') and loss tangent (tan δ) of AZ-containing homopolymer PM11AZC4. Frequency sweeps of (A) trans-rich PM11AZC4 and (B) cis-rich PM11AZC4, respectively. Temperature sweeps of (C) trans-rich PM11AZC4 and (D) cis-rich PM11AZC4.

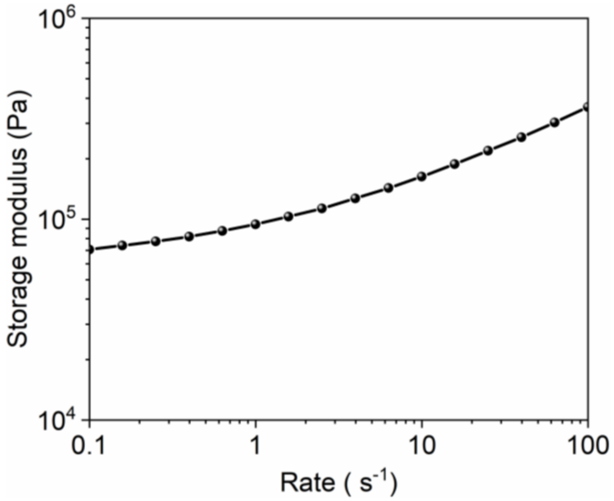

Fig. S19.
The rate-dependency modulus of cis-rich TBC film.

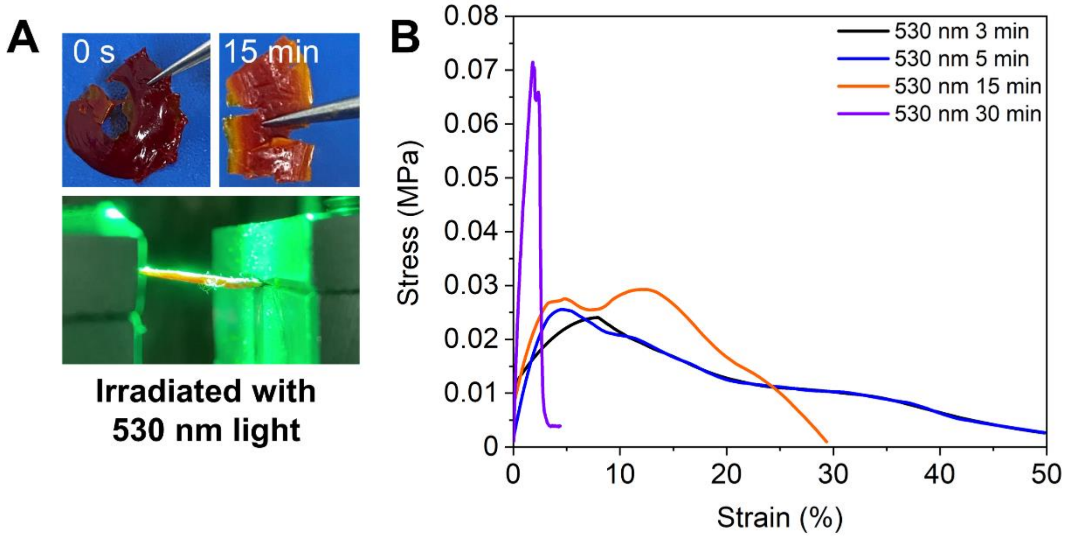

Fig. S20.
The cis-rich TBC film irradiated with 530 nm light. (A) The optical images of the cis-rich TBC film at initial state and the cis-rich TBC film after being irradiated with 530 nm light for 15 min. (B) The stress-strain curves of cis-rich TBC films being irradiated in-situ with 530 nm light for different time.

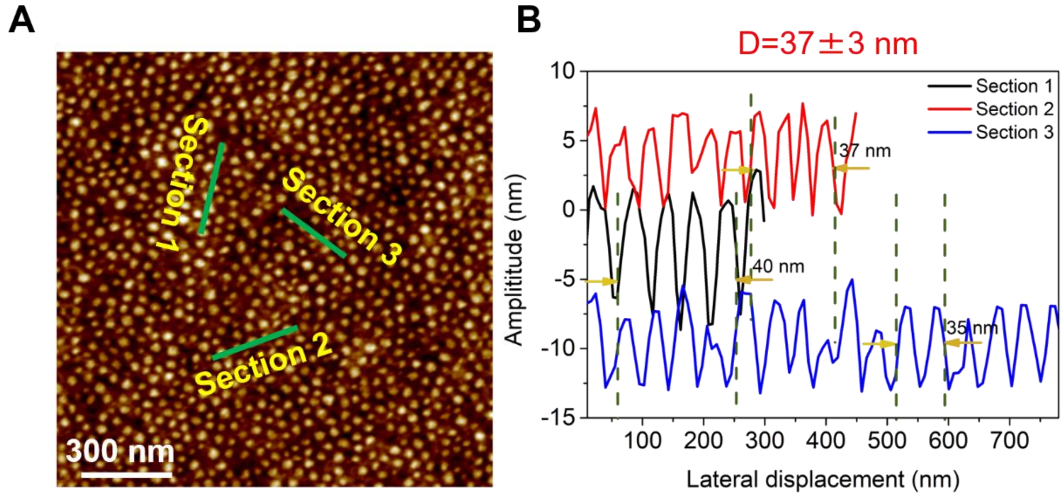

Fig. S21.
The MPS nanostructure characterization of the TBC film. (A) AFM phase image of the TBC film after thermal annealing. (B) The relationship between amplitude and displacement on different sections of AFM phase image.

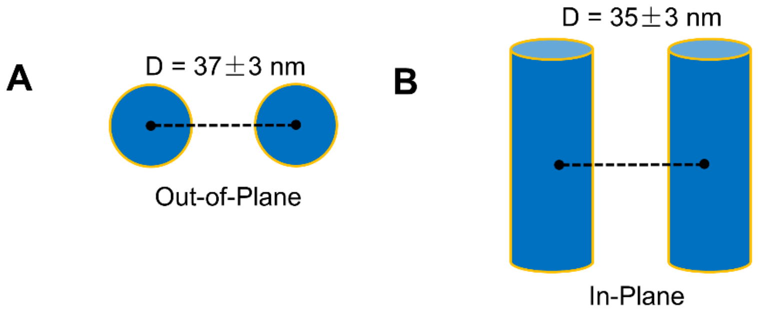

Fig. S22.
The orientation of cylindrical PS microdomains of the TBC film. Schematic representation of the orientation of cylindrical PS microdomains of the TBC film (A) after annealing, and (B) after imprinting with grating PDMS mould.

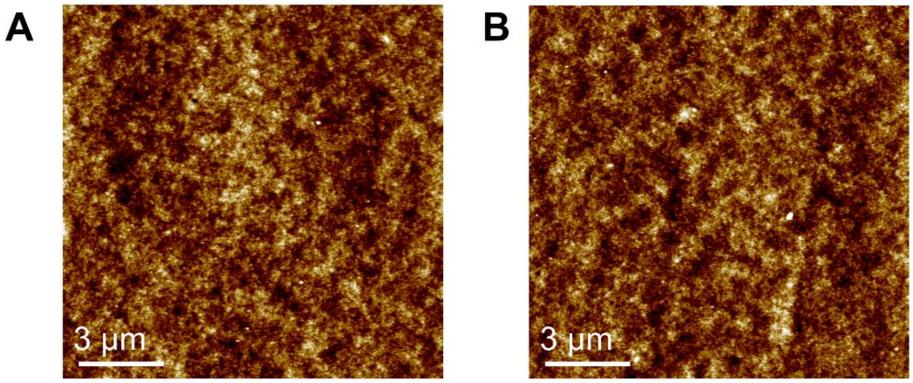

Fig. S23.
The nanopattern imprinting with TBC film. (A) AFM height image of the as-cast TBC film. (B) AFM height image of the TBC film pressed with an applied mechanical stress (1.35×10^5 Pa) at dark environment.

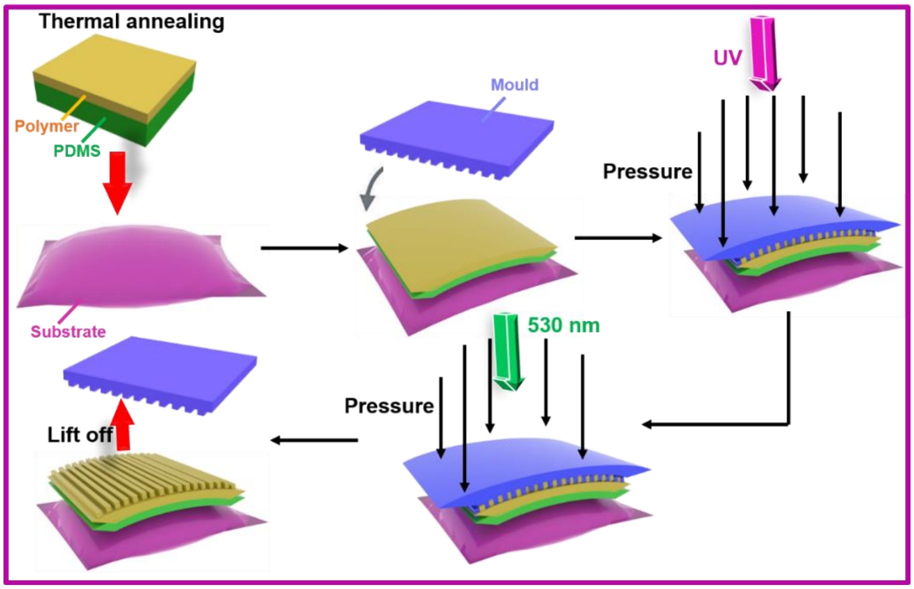

Fig. S24.

The schematic illustration of nonplanar nanopattern imprinting includes: thermal annealing; imprinting; 365 nm UV irradiation; and solidification with 530 nm visible light.

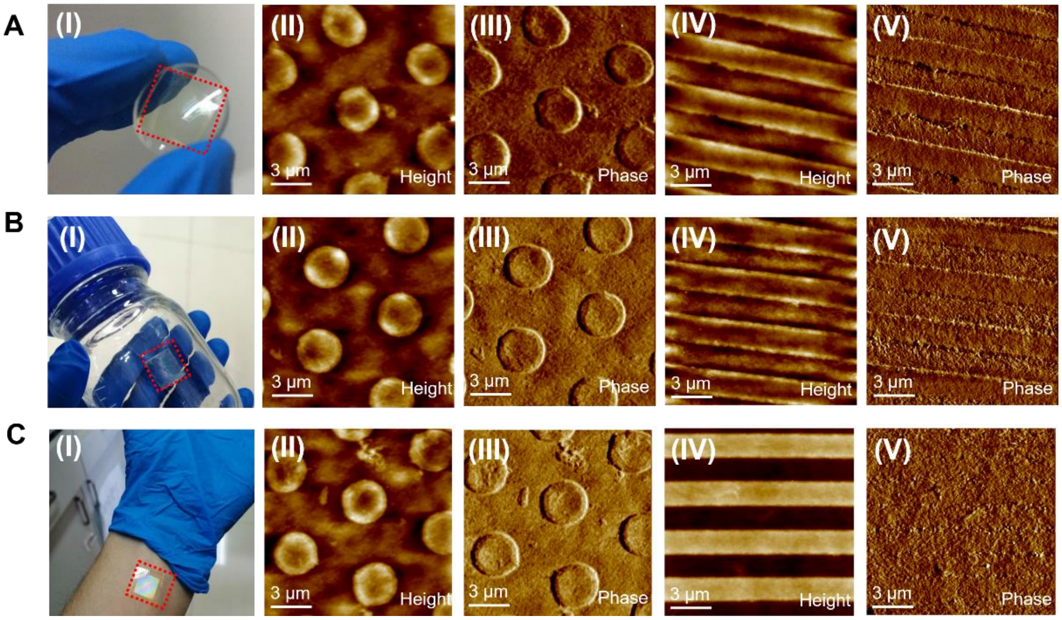

Fig. S25.

The photographs and AFM images of the nanopattern imprinting with TBC films. (A) The nanopattern imprinting on convex lens. (B) The nanopattern imprinting on sample bottle. (C) The nanopattern imprinting on the forearm of a person.

Table S1.
Comparison of DMA-measured storage modulus (G'), loss modulus (G'') and loss tangent (tan δ) of TBC and homopolymer PM11AZC4 at frequency (F) mode.

Samples	Trans-rich		Cis-rich	
	G'	tanδ	G'	tanδ
TBC	Moduli: 10^7-10^8	tanδ<1	Moduli: 10^4-10^6	tanδ<1
PM11AZC4	Moduli: 10^7-10^8	tanδ<1	Moduli: 10^3-10^6	tanδ>1

Table S2.
Comparison of DMA-measured loss tangent (tan δ) of TBC and homopolymer PM11AZC4 at temperature mode.

Samples	Trans-rich	Cis-rich
	tanδ	tanδ
TBC	tanδ<1	tanδ<1
PM11AZC4	tanδ<1	tanδ>1

Table S3.
Elasticity modulus and elongation at break of cis-rich TBC films irradiated with 530 nm light for different time.

Cis film	530 nm 0 s	530 nm 5 min	530 nm 15 min	530 nm 30 min
Elasticity modulus (MPa)	0.2	1.02	1.45	5.97
Elongation at break (%)	125%	49%	29%	3%

Movie S1.
Mechanical behavior of trans-rich TBC film.

Movie S2.
Mechanical behavior of cis-rich TBC film.

Movie S3.
The stretching and recovery of cis-rich TBC film.

Movie S4.
The flexible display device on the forearm.

Movie S5.
The flexible display device rotated at different angles.

Movie S6.
The naked and packaged perovskite solar cells immersed in water.

REFERENCES AND NOTES

1. H. Yuk, T. Zhang, G. A. Parada, X. Liu, X. Zhao, Skin-inspired hydrogel-elastomer hybrids with robust interfaces and functional microstructures. *Nat. Commun.* **7**, 12028 (2016).

2. Y. Wang, C. Zhao, J. Wang, X. Luo, L. Xie, S. Zhan, J. Kim, X. Wang, X. Liu, Y. Ying, Wearable plasmonic-metasurface sensor for noninvasive and universal molecular fingerprint detection on biointerfaces. *Sci. Adv.* **7**, eabe4553 (2021).

3. H. Jinno, K. Fukuda, X. Xu, S. Park, Y. Suzuki, M. Koizumi, T. Yokota, I. Osaka, K. Takimiya, T. Someya, Stretchable and waterproof elastomer-coated organic photovoltaics for washable electronic textile applications. *Nat. Energy* **2**, 780–785 (2017).

4. G. S. Jeong, D. H. Baek, H. C. Jung, J. H. Song, J. H. Moon, S. W. Hong, I. Y. Kim, S. H. Lee, Solderable and electroplatable flexible electronic circuit on a porous stretchable elastomer. *Nat. Commun.* **3**, 977 (2012).

5. R. Xie, S. Mukherjee, A. E. Levi, V. G. Reynolds, H. Wang, M. L. Chabinyc, C. M. Bates, Room temperature 3D printing of super-soft and solvent-free elastomers. *Sci. Adv.* **6**, eabc6900 (2020).

6. G. Chen, Y. Zhao, Y. Fang, X. Zhao, S. Shen, T. Tat, A. Nashalian, J. Chen, Wearable ultrahigh current power source based on giant magnetoelastic effect in soft elastomer system. *ACS Nano* **15**, 20582–20589 (2021).

7. O. Unver, M. Sitti, Tankbot: A palm-size, tank-like climbing robot using soft elastomer adhesive treads. *Ind. Robot.* **29**, 1761–1777 (2010).

8. H. Guo, Y. Han, W. Zhao, J. Yang, L. Zhang, Universally autonomous self-healing elastomer with high stretchability. *Nat. Commun.* **11**, 2037 (2020).

9. Y. Alapan, A. C. Karacakol, S. N. Guzelhan, I. Isik, M. Sitti, Reprogrammable shape morphing of magnetic soft machines. *Sci. Adv.* **6**, eabc6414 (2020).

10. P. Hu, J. Madsen, A. L. Skov, One reaction to make highly stretchable or extremely soft silicone elastomers from easily available materials. *Nat. Commun.* **13**, 370 (2022).

11. A. Lendlein, R. Langer, Biodegradable, elastic shape-memory polymers for potential biomedical applications. *Science* **296**, 1673–1676 (2002).

12. C. C. Honeker, E. L. Thomas, Impact of morphological orientation in determining mechanical properties in triblock copolymer systems. *Chem. Mater.* **8**, 1702–1714 (1996).

13. A. Takano, I. Kamaya, Y. Takahashi, Y. Matsushita, Effect of loop/bridge conformation ratio on elastic properties of the sphere-forming ABA triblock copolymers: Preparation of samples and determination of loop/bridge ratio. *Macromolecules* **38**, 9718–9723 (2005).

14. M. Hayashi, S. Matsushima, A. Noro, Y. Matsushita, Mechanical property enhancement of ABA block copolymer-based elastomers by incorporating transient cross-links into soft middle block. *Macromolecules* **48**, 421–431 (2015).

15. D. Yamaguchi, M. Cloitre, P. Panine, L. Leibler, Phase behavior and viscoelastic properties of thermoplastic elastomer gels based on ABC triblock copolymers. *Macromolecules* **38**, 7798–7806 (2005).

16. S. Wang, S. V. Kesava, E. D. Gomez, M. L. Robertson, Sustainable thermoplastic elastomers derived from fatty acids. *Macromolecules* **46**, 7202–7212 (2013).

17. D. D. Han, Y. L. Zhang, J. N. Ma, Y. Q. Liu, B. Han, H. B. Sun, Light-mediated manufacture and manipulation of actuators. *Adv. Mater.* **28**, 8328–8343 (2016).

18. F. Cai, F. Zheng, X. Lu, Q. Lu, Control of the alignment of liquid crystal molecules on a sequence-polymerized film by surface migration and polarized light irradiation. *Polym. Chem.* **8**, 7316–7324 (2017).

19. X. Lv, W. Wang, A. J. Clancy, H. Yu, High-speed, heavy-load, and direction-controllable photothermal pneumatic floating robot. *ACS Appl. Mater. Interfaces* **13**, 23030–23037 (2021).

20. X. Lv, M. Yu, W. Wang, H. Yu, Photothermal pneumatic wheel with high loadbearing capacity. *Compos. Commun.* **24**, 100651 (2021).

21. P. Zhang, B. Wu, S. Huang, F. Cai, H. Yu, UV-vis-NIR light-induced bending of shape-memory polyurethane composites doped with azobenzene and upconversion nanoparticles. *Polymer* **178**, 121644 (2019).

22. H. K. Bisoyi, Q. Li, Light-driven liquid crystalline materials: From photo-induced phase transitions and property modulations to applications. *Chem. Rev.* **116**, 15089–15166 (2016).

23. Y. Tian, K. Watanabe, X. Kong, J. Abe, T. Iyoda, Synthesis, nanostructures, and functionality of amphiphilic liquid crystalline block copolymers with azobenzene moieties. *Macromolecules* **35**, 3739–3747 (2002).

24. H. Yu, T. Kobayashi, H. Yang, Liquid-crystalline ordering helps block copolymer self-assembly. *Adv. Mater.* **23**, 3337–3344 (2011).

25. W. Mu, Z. Ji, M. Zhou, J. Wu, Y. Lin, Y. Qiao, Membrane-confined liquid-liquid phase separation toward artificial organelles. *Sci. Adv.* **7**, eabf9000 (2021).

26. S. Nagano, Y. Koizuka, T. Murase, M. Sano, Y. Shinohara, Synergy effect on morphology switching: Real-time observation of photo-orientation of microphase separation in a block copolymer. *Angew. Chem. Int. Ed.* **124**, 5986–5990 (2012).

27. M. Sano, F. Shan, M. Hara, S. Nagano, Y. Shinohara, Y. Amemiya, T. Seki, Dynamic photoinduced realignment processes in photoresponsive block copolymer films: Effects of the chain length and block copolymer architecture. *Soft Matter* **11**, 5918–5925 (2015).

28. S. Ito, H. Akiyama, R. Sekizawa, M. Mori, M. Yoshida, H. Kihara, Light-induced reworkable adhesives based on ABA-type triblock copolymers with azopolymer termini. *ACS Appl. Mater. Interfaces* **10**, 32649–32658 (2018).

29. Z. S. Davidson, H. Shahsavan, A. Aghakhani, Y. Guo, L. Hines, Y. Xia, S. Yang, M. Sitti, Monolithic shape-programmable dielectric liquid crystal elastomer actuators. *Sci. Adv.* **5**, eaay0855 (2019).

30. H. A. Haque, M. Hara, S. Nagano, T. Seki, Photoinduced in-plane motions of azobenzene mesogens affected by the flexibility of underlying amorphous chains. *Macromolecules* **46**, 8275–8283 (2013).

31. S. Lee, J. Shin, Y. H. Lee, S. Fan, J. K. Park, Directional photofluidization lithography for nanoarchitectures with controlled shapes and sizes. *Nano Lett.* **10**, 296–304 (2010).

32. K. Hisano, M. Aizawa, M. Ishizu, Y. Kurata, W. Nakano, N. Akamatsu, C. J. Barrett, A. Shishido, Scanning wave photopolymerization enables dye-free alignment patterning of liquid crystals. *Sci. Adv.* **3**, e1701610 (2017).

33. B. Yang, M. Yu, H. Yu, Azopolymer-based nanoimprint lithography: Recent developments in methodology and applications. *ChemPlusChem* **85**, 2166–2176 (2020).

34. B. Yang, F. Cai, S. Huang, H. Yu, Athermal and soft multi-nanopatterning of azopolymers: Phototunable mechanical properties. *Angew. Chem. Int. Ed.* **59**, 4035–4042. (2020).

35. H. W. Zhou, C. G. Xue, P. Weis, Y. Suzuki, S. L. Huang, K. Koynov, G. K. Auernhammer, R. Berger, H. J. Butt, S. Wu, Photoswitching of glass transition temperatures of azobenzene-containing polymers induces reversible solid-to-liquid transitions. *Nat. Chem.* **9**,145–151 (2017).

36. P. Weis, W. Tian, S. Wu, Photoinduced liquefaction of azobenzene-containing polymers. *Chem. A Eur. J.* **24**, 6494–6505 (2018).

37. S. Bai, Y. Zhao, Azobenzene-containing thermoplastic elastomers: Coupling mechanical and optical effects. *Macromolecules* **34**, 9032–9038 (2001).

38. L. Cui, X. Tong, X. Yan, G. Liu, Y. Zhao, Photoactive thermoplastic elastomers of azobenzene-containing triblock copolymers prepared through atom transfer radical polymerization. *Macromolecules* **37**, 7097–7104 (2004).

39. L. Yin, L. Han, F. Ge, X. Tong, W. Zhang, A. Soldera, Y. Zhao, A novel side-chain liquid crystal elastomer exhibiting anomalous reversible shape change. *Angew. Chem. Int. Ed.* **132**, 15241–15246 (2020).

40. T. Ikeda, O. Tsutsumi, Optical switching and image storage by means of azobenzene liquid-crystal films. *Science* **268**, 1873–1875 (1995).

41. W. C. Xu, S. Sun, S. Wu, Photoinduced reversible solid-to-liquid transitions for photoswitchable materials. *Angew. Chem. Int. Ed.* **58**, 9712–9740 (2019).

42. F. Cai, T. Song, B. Yang, X. Lv, L. Zhang, H. Yu, Enhancement of solar thermal fuel by microphase separation and nanoconfinement of a block copolymer. *Chem. Mater.* **33**, 9750–9759 (2021).

43. Y. Matsumiya, H. Watanabe, A. Takano, Y. Takahashi, Uniaxial extensional behavior of (SIS)p-type multiblock copolymer systems: Structural origin of high extensibility. *Macromolecules* **46**, 2681–2695 (2013).

44. Yi, X. Fan, X. Wan, L. Li, N. Zhao, X. Chen, J. Xu, Q.-F. Zhou, ABA type triblock copolymer based on mesogen-jacketed liquid crystalline polymer: Design, synthesis, and potential as thermoplastic elastomer. *Macromolecules* **37**, 7610–7618 (2004).

45. Y. Chen, S. Tan, N. Li, B. Huang, X. Niu, L. Li, M. Sun, Y. Zhang, X. Zhang, C. Zhu, N. Yang, H. Zai, Y. Wu, S. Ma, Y. Bai, Q. Chen, F. Xiao, K. Sun, H. Zhou, Self-elimination of intrinsic defects improves the low-temperature performance of perovskite photovoltaics. *Joule* **4**, 1961–1976 (2020).

46. T. Wang, X. Li, Z. Dong, S. Huang, H. Yu, Vertical orientation of nanocylinders in liquid-crystalline block copolymers directed by light. *ACS Appl. Mater. Interfaces* **9**, 24864–24872 (2017).

47. V. Czikklely, H. D. Forsterling, H. Kuhn, Extended dipole model for aggregates of dye molecules. *Chem. Phys. Lett.* **6**, 207–210 (1970).

48. H. M. D. Bandara, S. C. Burdette, Photoisomerization in different classes of azobenzene. *Chem. Soc. Rev.* **41**, 1809–1825 (2012).

49. Y. Chen, S. Huang, T. Wang, Z. Dong, H. Yu, Confined self-assembly enables stabilization and patterning of nanostructures in liquid-crystalline block copolymers. *Macromolecules* **52**, 1892–1898 (2019).
